# Global Habitat Suitability Modeling of the Giant Honeybee (*Apis dorsata*) Under Future Climate Change Scenarios

**DOI:** 10.3390/insects17090954

**Published:** 2026-09-12

**Authors:** Xinjian Xu, Shujing Zhou, Jiangpeng Li, Xiangjie Zhu, Hossam F. Abou-Shaara

**Affiliations:** 1 College of Bee Science, Fujian Agriculture and Forestry University, Fuzhou 350002, China; xuxinjian1982@fafu.edu.cn (X.X.); sjzhou118@fafu.edu.cn (S.Z.); 52306009011@fafu.edu.cn (J.L.); 2 Department of Plant Protection, Faculty of Agriculture, Damanhour University, Damanhour 22516, Egypt

**Keywords:** habitat suitability, modeling, climate change, MaxEnt, *Apis dorsata*

## Abstract

The giant honeybee, *Apis dorsata*, is an important pollinator native to tropical and subtropical Asia. Understanding its potential response to climate change is important for pollinator conservation, ecological risk assessment, and biosecurity planning. This study used an optimized MaxEnt ecological niche modeling framework to predict the current and future global habitat suitability of *A. dorsata*. The model was developed using 1060 occurrence records and seven non-collinear bioclimatic variables and projected under three global climate models (IPSL-CM6A-LR, BCC-CSM2-MR, and MPI-ESM1-2-HR) and three Shared Socioeconomic Pathways (SSP126, SSP245, and SSP585) for 2041–2060, centered on 2050. The optimized model used linear, quadratic, and hinge features (LQH) with a regularization multiplier of 0.5 and demonstrated good predictive performance under 5-fold spatial cross-validation (mean AUC = 0.905 ± 0.007; TSS = 0.746 ± 0.010). The results indicate that the potential distribution of *A. dorsata* is primarily associated with the combined effects of seasonal temperature and moisture availability. Current projections identified high climatic suitability across South and Southeast Asia, while also revealing potentially suitable environments in parts of Africa, the Americas, and northern Australia. Future projections suggest that suitable climatic conditions will largely persist through 2050, with habitat gains generally exceeding losses and increasing under higher climate-forcing scenarios. Continued monitoring and proactive biosecurity are essential to address habitat loss within the native range and prevent establishment in newly suitable regions. This study highlights the potential redistribution of *A. dorsata* under future climate change.

## Simple Summary

This study used an optimized MaxEnt model to predict the current and future global habitat suitability of the giant honeybee (*Apis dorsata*), an important pollinator native to South and Southeast Asia. The model showed good predictive performance. Habitat suitability was primarily influenced by the combined effects of seasonal temperature and moisture availability. Current projections indicate that highly suitable habitats are concentrated within the species’ native range, while also identifying potentially suitable areas in parts of Africa, the Americas, and northern Australia. Future climate projections suggest that suitable habitats will largely persist and expand, with habitat gains generally exceeding losses.

## 1. Introduction

Wild honeybees play a vital role in sustaining global biodiversity and agricultural productivity, serving as keystone pollinators that ensure the reproductive success of numerous angiosperms and provide high-value ecosystem services [1,2,3]. Among these, the giant honeybee, *Apis dorsata* Fabricius 1793 (Hymenoptera: Apidae), is a prominent open-nesting species native to South and Southeast Asia, renowned for its significant contributions to crop pollination [2,4]. Unlike managed species such as *A. mellifera*, *A. dorsata* is highly migratory, undertaking complex seasonal movements across diverse habitats, including mangrove forests and high-elevation Himalayan foothills, to optimize resource exploitation [2,3]. These movements are primarily linked to seasonal patterns of floral-resource availability, including mass flowering events in forest trees and mangrove vegetation, which may be influenced by variations in temperature and precipitation [2,3,4]. This species constructs massive, single-comb nests on cliffs, tall trees, or anthropogenic structures, yielding between 50 and 80 kg of honey per colony [5,6]. Colonies maintain a sophisticated defensive posture, utilizing “shimmering” waves to deter predators, even during periods of mass flight activity [4,7]. However, *A. dorsata* faces escalating threats from climate change, habitat fragmentation, and destructive honey-hunting practices, which have contributed to declines in natural populations and pose risks to local food security [3,6,8].

The native geographical range of *A. dorsata* traditionally extends from the Indian subcontinent to the Indonesian archipelago and the Philippines [6,9]. In recent years, however, its distribution patterns have shifted significantly in response to environmental pressures, with notable fluctuations in its occurrence across landscapes ranging from primary forests to urbanized centers [2,3]. These changes are of particular concern because *A. dorsata* supports regional biodiversity through the pollination of numerous wild flora and high-value timber species [10,11]. Factors influencing its contemporary distribution include habitat fragmentation and the loss of traditional nesting sites, while anthropogenic climate change and rising mean temperatures are considered among the most important drivers affecting migratory routes and long-term survival across its range [2,3,6].

Climate change continues to play a major role in reshaping species distributions and altering the ecological niches of essential pollinators [12,13,14]. As global temperatures continue to increase, wild bee populations are becoming increasingly vulnerable because these species are highly sensitive to changing environmental conditions and disruptions in floral phenology [3,15,16]. Recent evidence suggests that the synergy between climate warming and anthropogenic pressures, such as habitat fragmentation and land-use intensification, has significantly altered the population dynamics of the genus *Apis* [13,17]. Recent modeling indicates that in regions like Pakistan and the Himalayan foothills, up to 79% of suitable habitat for *A. dorsata* could disappear under extreme climate scenarios by the late 21st century [2]. This decline poses a substantial threat to local biodiversity and agricultural stability, specifically through the loss of keystone pollination services and the potential disruption of indigenous honey-harvesting economies. While the species possesses some migratory plasticity to track seasonal resources, its limited adaptability compared to managed bees makes it particularly vulnerable to rapid environmental shifts [2,3,6]. Despite these documented ecological risks, the potential future distribution of *A. dorsata* under various socioeconomic pathways remains insufficiently explored.

Species distribution models (SDMs) are a valuable toolset for assessing climate change impacts on pollinator ranges and the ecological functions they support. Among the available algorithms, Maximum Entropy (MaxEnt) is widely recognized as a tool for predicting species distributions [18,19,20]. The efficacy of MaxEnt lies in its ability to handle complex environmental interactions and small sample sizes, making it highly reliable for assessing the habitat suitability of bee species in rapidly changing landscapes [21,22,23]. Given the limited understanding of the potential global expansion of *A. dorsata*, this study provides a novel contribution to the field. This study evaluates the climatic suitability of the giant honeybee, *A. dorsata*, based on temperature and precipitation and projects potential range shifts by 2050 under different Shared Socioeconomic Pathway (SSP) scenarios. Identifying areas of habitat loss, persistence, and potential refugia is essential for developing proactive conservation and adaptive management strategies to safeguard wild honey resources and tropical forest ecosystems. Moreover, identifying climatically suitable areas outside the native range provides a valuable basis for ecological monitoring and biosecurity planning, enabling targeted surveillance and early detection to help prevent unintended establishment. Ultimately, this study advances our understanding of how bioclimatic factors shape the current and future distribution of *A. dorsata*.

## 2. Materials and Methods

### 2.1. Occurrence Data Processing and Spatial Thinning

Occurrence records for *A. dorsata* were retrieved from the Global Biodiversity Information Facility (GBIF). The dataset comprised research-grade observations sourced from iNaturalist (GBIF.org GBIF Occurrence Download. Available online: https://doi.org/10.15468/dl.w4vkg8 (accessed on 7 April 2026)). The basis of record was restricted to human observations to verify the physical presence of *A. dorsata* at each recorded location. The temporal scope was limited to observations recorded between January 2020 and April 2026, ensuring that only recent records were included. The initial download yielded 6342 records, all with an occurrence status of “present”. To ensure data integrity, a rigorous cleaning protocol was implemented using R (version 4.5.1). The analysis was conducted using a combination of packages, including tidyverse for data preprocessing, sf for spatial data processing, CoordinateCleaner for coordinate validation, spThin for spatial thinning, skimr for descriptive data exploration, and rnaturalearth and rnaturalearthdata for obtaining global geographic layers.

Initially, the data were visually checked to ensure that *A. dorsata* was the scientific name recorded for each observation and that geographic coordinates were available. Records that did not meet these criteria were removed, resulting in a total of 5918 records. During the second stage of data cleaning, longitude and latitude values were converted to numeric format, and records with incomplete or missing geographic coordinates were removed. Of the 5918 records, 5916 records had complete coordinate information and were retained. Exact duplicate records were subsequently removed. This step reduced the dataset to 5290 unique occurrence records. This procedure was applied to minimize redundant sampling at identical geographic locations.

The geographic validity of the remaining records was assessed using the CoordinateCleaner package. The validation procedure incorporated tests for invalid coordinate values, zero coordinates, equal or duplicated latitude and longitude values, and occurrences located in the sea. Records passing the CoordinateCleaner validation were retained for further analysis. In this dataset, all 5290 records passed the applied coordinate validation criteria, indicating that no additional records were removed at this stage. The validated records were then converted into a spatial feature object using the WGS84 geographic coordinate reference system.

To define the geographic extent of the analysis, records located in the polar regions were excluded. Specifically, occurrences north of 66.5° N were considered part of the Arctic region and occurrences south of 60° S were considered part of Antarctica; these records were excluded from subsequent analyses. No records in the dataset fell outside these latitudinal limits, so the dataset remained at 5290 records after this filtering step. A further geographic filtering procedure was then performed to retain only occurrences located on land. A global Natural Earth land polygon layer was used as the reference geographic boundary, and spatial intersection was performed between the occurrence points and the land polygons. Records falling outside the land polygons, and therefore representing oceanic locations, were removed. This procedure reduced the dataset from 5290 to 5013 records.

To reduce potential spatial sampling bias caused by clusters of geographically close occurrence records, spatial thinning was performed using the spThin package. A minimum distance of 20 km was specified between retained occurrence records, with one thinning replicate performed. A fixed random seed (123) was used to ensure that the thinning procedure was reproducible. The spatial thinning procedure substantially reduced the dataset from 5013 land-based records to 1060 occurrence records (Supplementary Materials; File S1). The resulting records were used as the final cleaned and spatially thinned occurrence dataset.

**Figure 1:**
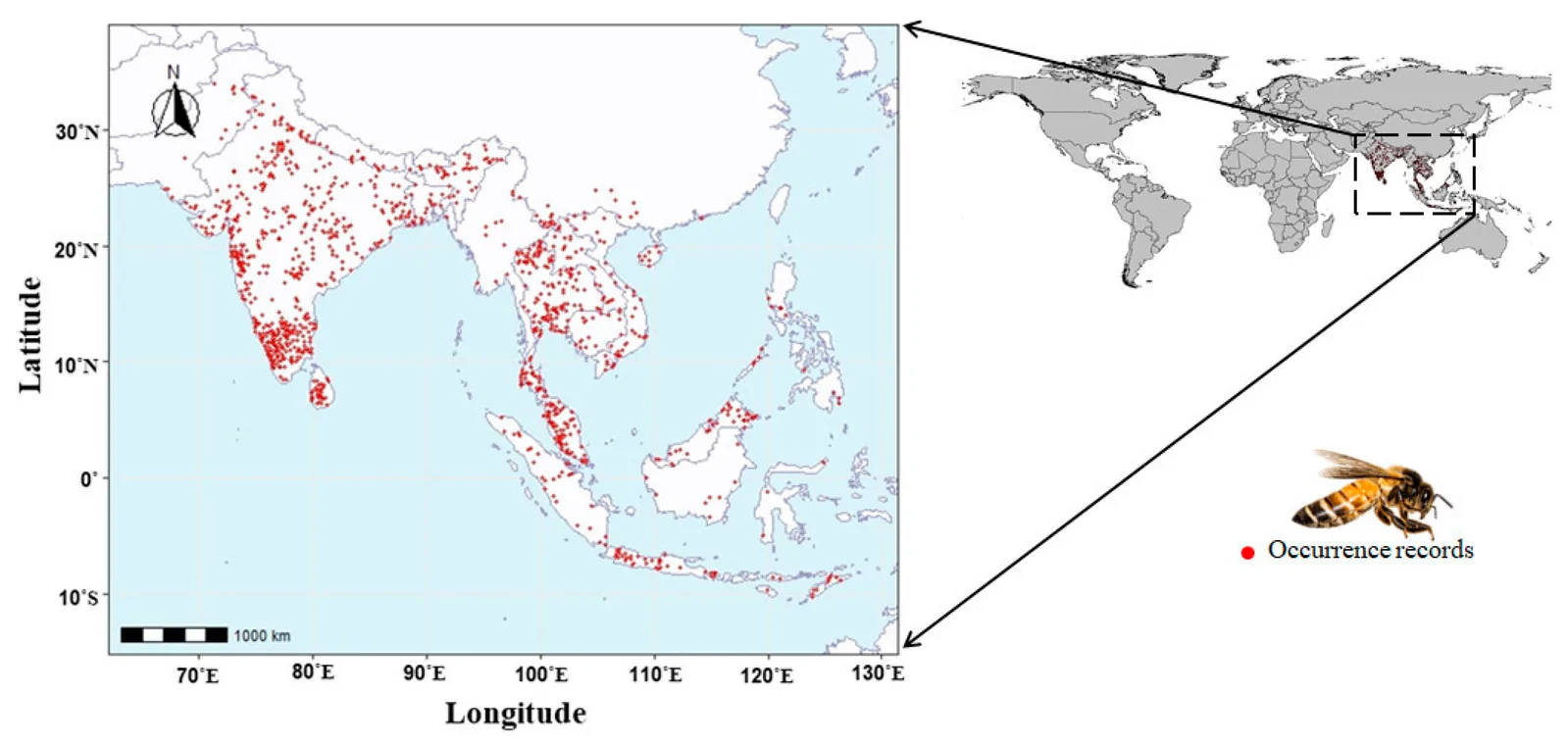
Presence records of *Apis dorsata* used in the study, comprising a total of 1060 occurrence records.

The spatial distribution of the cleaned occurrence records was visualized on a global map using country boundaries obtained from Natural Earth. The final 1060 occurrences were plotted according to their geographic coordinates (Figure 1).

### 2.2. Environmental Data Preparation and Definition of the Accessible Area (M)

The final occurrence dataset obtained after geographic cleaning and 20 km spatial thinning was used as the input occurrence dataset. Species distribution modelling (SDM) was conducted in R (version 4.5.1) using the MaxEnt algorithm implemented through the maxnet package, with model calibration and evaluation supported by the ENMeval framework. Additional packages, including terra, usdm, dismo, ecospat, blockCV, sf, ggplot2, and pROC, were used for environmental raster processing, multicollinearity assessment, spatial analysis, model evaluation, and visualization (Supplementary Materials; File S2). The modelling workflow distinguished between the accessible area (*M*) used for model calibration and a broader global area used for model projection.

Environmental predictors were obtained from the WorldClim version 2.1 BioClim dataset at a spatial resolution of 10 arc-minutes [24]. Raster files corresponding to the 19 standard bioclimatic variables (bio1–bio19) were imported into R and arranged numerically according to their BioClim layer numbers. All 19 variables were retained during the initial environmental data preparation stage. A global projection extent was defined from 180° W to 180° E and from 60° S to 66.5° N, thereby excluding Antarctica and the Arctic region. All 19 bioclimatic raster layers were cropped to this extent to generate the environmental raster stack used for subsequent global model projection.

To define the geographic area potentially accessible to the species, a combined accessible area (*M*) was delineated using two complementary components. First, a 100-km buffer was generated around each occurrence record. Buffering was performed after transforming the occurrence points to an equal-area coordinate reference system. Individual buffers were subsequently merged into a single geographic region, transformed back to WGS84, and restricted to terrestrial environments by intersecting the resulting area with a global land polygon.

Second, the entire continental extent of Asia was included as an additional component of the accessible area. Countries classified as belonging to Asia were extracted from the global land dataset and merged to create a unified Asian terrestrial region. The land-restricted 100-km occurrence-buffer area and the Asian continental extent were then combined using a spatial union operation. The resulting combined region was intersected with the global terrestrial land area to remove marine environments and generate the final accessible area (*M*) (Supplementary Figure S1; File S3).

The WorldClim bioclimatic raster layers were subsequently cropped and masked to the final combined *M* region, producing the environmental raster stack used for model calibration. This approach allowed model calibration to consider both the immediate geographic surroundings of known occurrences and the wider Asian region considered potentially accessible to the species, while excluding marine environments and areas outside the defined modelling extent.

### 2.3. Multicollinearity Assessment and Environmental Variable Selection

Prior to model calibration, the 19 BioClim variables were evaluated for multicollinearity within the accessible area. Environmental values were extracted from the *M* raster stack at the occurrence locations. Pairwise Pearson’s correlations were calculated among all BioClim variables, and the resulting relationships were visualized using a correlation heatmap (Supplementary Figure S2; File S3). To further reduce redundancy among environmental predictors, correlation-based variance inflation filtering was performed using the vifcor function in the usdm package with a correlation threshold of r = 0.70. Variables exhibiting strong correlations above this threshold were excluded, thereby reducing the risk that highly correlated predictors would have disproportionate effects on model calibration and interpretation.

Following correlation-based filtering, seven climatic variables were retained for model development: bio2 (mean diurnal range), bio8 (mean temperature of the wettest quarter), bio9 (mean temperature of the driest quarter), bio13 (precipitation of the wettest month), bio14 (precipitation of the driest month), bio18 (precipitation of the warmest quarter), and bio19 (precipitation of the coldest quarter). A second Pearson’s correlation matrix was calculated and visualized for the selected variables to confirm the reduced level of correlation among the final predictors (Supplementary Figure S3; File S3). The seven selected variables were subsequently retained from both the environmental raster stack representing the accessible area (*M*) and the global environmental raster stack. The *M* stack was used for model calibration, whereas the corresponding global stack was retained for model projection. This ensured that model calibration and global projection were based on an identical set of selected, non-redundant climatic predictors.

### 2.4. Spatial Cross-Validation and MaxEnt Model Optimization

The resulting modelling framework therefore consisted of two complementary environmental domains: the accessible area (*M*) for model calibration and the global extent excluding the Arctic and Antarctica for spatial projection. Model calibration and optimization were conducted exclusively within the accessible area (*M*), whereas the global environmental stack containing the same selected climatic predictors was retained for subsequent spatial projection.

To account for spatial autocorrelation in the occurrence data and to generate spatially independent training and testing datasets, spatial block cross-validation was implemented using the cv_spatial function in the blockCV package. Prior to spatial partitioning, 10,000 background points were randomly sampled from the selected environmental raster stack within *M* using the spatSample function. Cells containing missing environmental values were excluded during background sampling. A fixed random seed of 42 was used to ensure reproducibility. The environmental raster stack and occurrence records were projected to the Web Mercator coordinate reference system for spatial block construction. Five spatially separated cross-validation folds were generated using spatial blocks with a size of 600 km. The spatial partitioning was generated using random block selection, with 100 iterations evaluated to obtain the final partitioning. The resulting folds contained between 192 and 248 occurrence records in the respective testing datasets, while the corresponding training datasets contained between 812 and 868 occurrence records. Background points were assigned to the same spatial cross-validation framework by identifying the spatial block intersecting each background location and assigning the corresponding block fold.

Model tuning was performed within the accessible area (*M*) using the ENMeval framework. The cleaned occurrence records, 10,000 background locations, and selected environmental predictors were used to calibrate and evaluate candidate models under the user-defined five-fold spatial partitioning. Two major MaxEnt tuning parameters were evaluated: feature class (FC) and regularization multiplier (RM). Three feature-class combinations were tested: L (linear), LQ (linear and quadratic), and LQH (linear, quadratic, and hinge features). Four regularization multiplier values (0.5, 1, 2, and 3) were evaluated for each feature-class combination, resulting in a total of 12 candidate model configurations.

Candidate models were compared using mean validation area under the receiver operating characteristic curve (AUC) and the change in Akaike Information Criterion corrected for small sample size (ΔAICc). Candidate models were ranked according to ΔAICc, and the model with the minimum ΔAICc value was selected as the optimal configuration. If ΔAICc values were unavailable, the model with the highest mean validation AUC was designated as the alternative selection criterion. The tuning procedure identified the LQH feature class with a regularization multiplier of 0.5 as the optimal model configuration, with ΔAICc = 0. This model achieved a mean validation AUC of 0.905. Therefore, the LQH feature-class combination and RM = 0.5 were selected for subsequent model fitting, prediction, and spatial evaluation (Supplementary Table S1; File S4).

### 2.5. Model Evaluation, Environmental Responses, and Variable Importance

The predictive performance of the optimized MaxEnt model was evaluated using the predefined five-fold spatial cross-validation framework. For each fold, occurrence and background records assigned to four spatial folds were used for model training, whereas occurrence and background records assigned to the remaining spatial fold were reserved for independent testing. Thus, both presence and background data were strictly partitioned according to the spatial block structure, reducing potential inflation of model performance caused by spatial dependence.

For each cross-validation iteration, the selected MaxEnt configuration, comprising LQH with RM of 0.5, was fitted using the maxnet package. Model predictions for the held-out occurrence and background records were generated using the complementary log-log (cloglog) output scale. Receiver operating characteristic (ROC) curves were then constructed by comparing predicted suitability values for testing occurrences with those for spatially independent testing background points.

For each fold, the area under the ROC curve (AUC), sensitivity, specificity, true skill statistic (TSS), continuous Boyce index (CBI), optimal suitability threshold, and omission rate were calculated. The optimal threshold, sensitivity, and specificity were obtained from the ROC curve using the criterion implemented by the coords function in the pROC package. TSS was calculated as sensitivity + specificity − 1, whereas the omission rate was calculated as the proportion of test occurrence records with predicted suitability values below the selected threshold.

The CBI was calculated using the ecospat package. For each fold, predicted suitability values at the test occurrence locations were compared with predictions obtained across the complete set of background locations. Model performance metrics were summarized across the five spatial folds by calculating the mean and standard error (SE).

Environmental response relationships were examined to assess changes in predicted suitability along gradients of each selected climatic variable. For each cross-validation fold, one predictor was varied across 100 equally spaced values spanning its observed range in the combined occurrence and background environmental dataset. All remaining predictors were held constant at their respective mean values. Predicted suitability was then calculated using the fold-specific MaxEnt model on the cloglog scale.

Response curves from the five cross-validation models were subsequently averaged for each environmental variable. The standard deviation among folds was calculated at each predictor value to represent variation in model responses. Mean response curves were presented together with uncertainty bands representing ±1 standard deviation, with predicted suitability constrained between 0 and 1.

Finally, a final MaxEnt model was fitted using all occurrence and background records with the optimized LQH feature classes and RM = 0.5. Baseline predictions were generated for the complete environmental dataset, and the corresponding AUC was calculated. Each environmental variable was then independently permuted while all other predictors remained unchanged, and model predictions and AUC values were recalculated. The reduction in AUC relative to the baseline model was used as an estimate of the importance of each predictor. Negative AUC reductions were set to zero. The resulting AUC reductions were standardized to percentages summing to 100%, thereby providing relative importance (%) of bioclimatic variables used in the MaxEnt model.

### 2.6. Global Habitat Suitability Projection

The final optimized MaxEnt model was fitted using all cleaned occurrence records and the sampled background data within the modelling framework, using the selected LQH feature classes and RM of 0.5. The final model was subsequently projected onto the global environmental raster stack to estimate potential habitat suitability across the broader modelling domain.

The global projection used the same seven selected, non-collinear BioClim variables retained during the multicollinearity assessment, thereby ensuring consistency between the environmental predictors used for model calibration and global projection. The projection covered the geographic extent from 180° W to 180° E and from 60° S to 66.5° N, excluding Antarctica and the Arctic region. Predictions were generated using the complementary log-log (cloglog) output scale, producing continuous habitat suitability values ranging from 0 to 1, with higher values indicating greater predicted environmental suitability for the species.

The continuous global suitability surface was visualized as a raster map, and the cleaned occurrence records were overlaid to facilitate comparison between known occurrence locations and areas predicted to have suitable environmental conditions.

For further interpretation and spatial quantification, the continuous suitability surface was classified into four habitat suitability categories using fixed threshold intervals. Suitability values from 0.00 to 0.25 were classified as rarely suitable, values greater than 0.25 to 0.50 as moderately suitable, values greater than 0.50 to 0.75 as highly suitable, and values greater than 0.75 to 1.00 as very highly suitable [18,19]. The categorized suitability map was also visualized with the cleaned occurrence records overlaid.

The geographic surface area represented by each suitability category was calculated using the terra package. Cell-specific surface areas were calculated using the cellSize function, accounting for variation in the actual surface area of raster cells across latitudes. The cell-area raster was then summarized according to the classified habitat suitability map using zonal statistics, with the areas of all cells belonging to each suitability category summed to obtain the total area represented by each class.

### 2.7. Future Habitat Suitability Projections

Future habitat suitability was projected for the 2041–2060 period, centered on 2050, under three Shared Socioeconomic Pathway (SSP) scenarios: SSP126, SSP245, and SSP585. Future climate projections from three Global Climate Models (GCMs)—IPSL-CM6A-LR, BCC-CSM2-MR, and MPI-ESM1-2-HR—were evaluated. The final optimized MaxEnt model, previously fitted using the selected LQH feature classes and RM of 0.5, was used for all future projections.

The same seven non-collinear BioClim variables selected during model development were used for future prediction. For each future climate dataset, the numerical BioClim indices corresponding to the selected baseline predictors were automatically identified and extracted. The extracted future layers were subsequently renamed to match the predictor names used during MaxEnt model calibration, thereby ensuring compatibility between the fitted model and future environmental datasets.

Future climate raster datasets were obtained from locally stored raster files and processed using the terra package. For each combination of GCM and SSP scenario, the relevant future BioClim layers were extracted and cropped to the predefined global modelling extent, extending from 180° W to 180° E and from 60° S to 66.5° N.

For each of the three GCMs under each of the three SSP scenarios, the optimized MaxEnt model was projected onto the corresponding future environmental conditions using the complementary log-log (cloglog) output scale. This resulted in nine individual GCM × SSP habitat suitability projections. Continuous predictions ranged from 0 to 1, with higher values representing greater predicted environmental suitability. Both continuous and categorized suitability maps were generated for each individual GCM–SSP combination.

To facilitate comparison among present and future projections, continuous suitability values were classified using the same four fixed suitability categories applied to the current-climate projection: rarely suitable (0.00–0.25), moderately suitable (>0.25–0.50), highly suitable (>0.50–0.75), and very highly suitable (>0.75–1.00).

For each SSP scenario, an ensemble prediction was generated by calculating the cell-by-cell arithmetic mean of the three corresponding GCM projections. This produced three SSP-level ensemble maps representing the average projected habitat suitability under SSP126, SSP245, and SSP585. The ensemble surfaces were generated on the continuous cloglog scale and subsequently classified using the same four suitability thresholds.

In addition to the SSP-specific ensembles, a grand overall future ensemble was generated by calculating the cell-by-cell mean across all nine individual GCM × SSP projections. This overall ensemble integrated variation among both climate models and emission scenarios and provided a generalized representation of projected habitat suitability under the full range of future climate conditions examined.

Surface area was calculated for each individual GCM × SSP projection, each SSP-level ensemble, and the grand overall future ensemble, resulting in a total of 13 projection summaries. For each continuous prediction raster, suitability values were first classified into the four predefined categories. Cell-specific surface areas were then calculated using the cellSize function in the terra package, accounting for geographic variation in raster-cell area.

Zonal statistics were subsequently applied to sum the areas of cells belonging to each suitability category. For every projection and ensemble, the total area of rarely suitable, moderately suitable, highly suitable, and very highly suitable habitat was calculated and expressed as a percentage of the total mapped area. This approach enabled quantitative comparisons of projected habitat suitability among individual climate models, emission scenarios, SSP-level ensembles, and the overall future climate ensemble.

### 2.8. Spatial Range-Shift Analysis

A spatial range-shift analysis was conducted to quantify potential changes in predicted climatically suitable areas between current conditions and projected future conditions for 2050. The current global habitat suitability map was used as the baseline and compared with future suitability projections generated under three Shared Socioeconomic Pathway (SSP) scenarios (SSP126, SSP245, and SSP585) using three Global Climate Models (GCMs): IPSL-CM6A-LR, BCC-CSM2-MR, and MPI-ESM1-2-HR.

The same optimized MaxEnt model and the seven selected non-collinear BioClim predictors used in the preceding modelling steps were applied to all future climate datasets. For each GCM–SSP combination, the relevant future BioClim layers were extracted according to their numerical indices, renamed to match the predictor names used by the fitted MaxEnt model, and cropped to the predefined global modelling extent extending from 180° W to 180°E and from 60° S to 66.5° N. Continuous future habitat suitability was then predicted using the complementary log-log (cloglog) output scale.

Individual suitability projections were generated for each of the nine GCM × SSP combinations. For each SSP, an ensemble prediction was subsequently calculated as the cell-by-cell arithmetic mean of the three corresponding GCM projections. A grand overall future ensemble was also generated by calculating the cell-by-cell mean across all nine individual GCM × SSP projections. Consequently, spatial range-shift analyses were conducted for a total of 13 future projections, comprising nine individual climate-model projections, three SSP-level ensemble projections, and one grand overall ensemble.

To allow direct comparison between current and future predictions, continuous suitability maps were converted into binary suitable and unsuitable classes using a threshold of 0.236, corresponding to the mean optimal threshold obtained from the five-fold spatial cross-validation. Grid cells with predicted suitability values greater than or equal to this threshold were classified as suitable, whereas cells with values below the threshold were classified as unsuitable. The same threshold was consistently applied to the current baseline and all future projections.

Spatial changes between current and future binary suitability maps were initially classified using raster map algebra into four potential categories: unsuitable, loss, gain, and stable suitable habitat. The change categories were initially encoded using the expression: current binary suitability + (2 × future binary suitability) + 1, which produced four numerical classes corresponding to unsuitable (1), loss (2), gain (3), and stable suitable habitat (4).

The surface area associated with each range-shift category was calculated using cell-specific area estimates. Raster cell areas were calculated using the cellSize function in the terra package, thereby accounting for geographic variation in cell area across latitude. The areas of cells belonging to each change category were summed, and the resulting area was expressed as a percentage of the total valid area included in each change map.

This procedure was repeated for all nine individual GCM × SSP projections, the three SSP-level ensemble projections, and the grand overall future ensemble. The resulting range-shift statistics enabled comparisons of projected habitat loss, gain, and persistence among individual climate models, emission scenarios, SSP-specific ensemble predictions, and the overall future climate ensemble.

## 3. Results

### 3.1. Predictive Performance and Model Discriminative Ability

**Figure 2:**
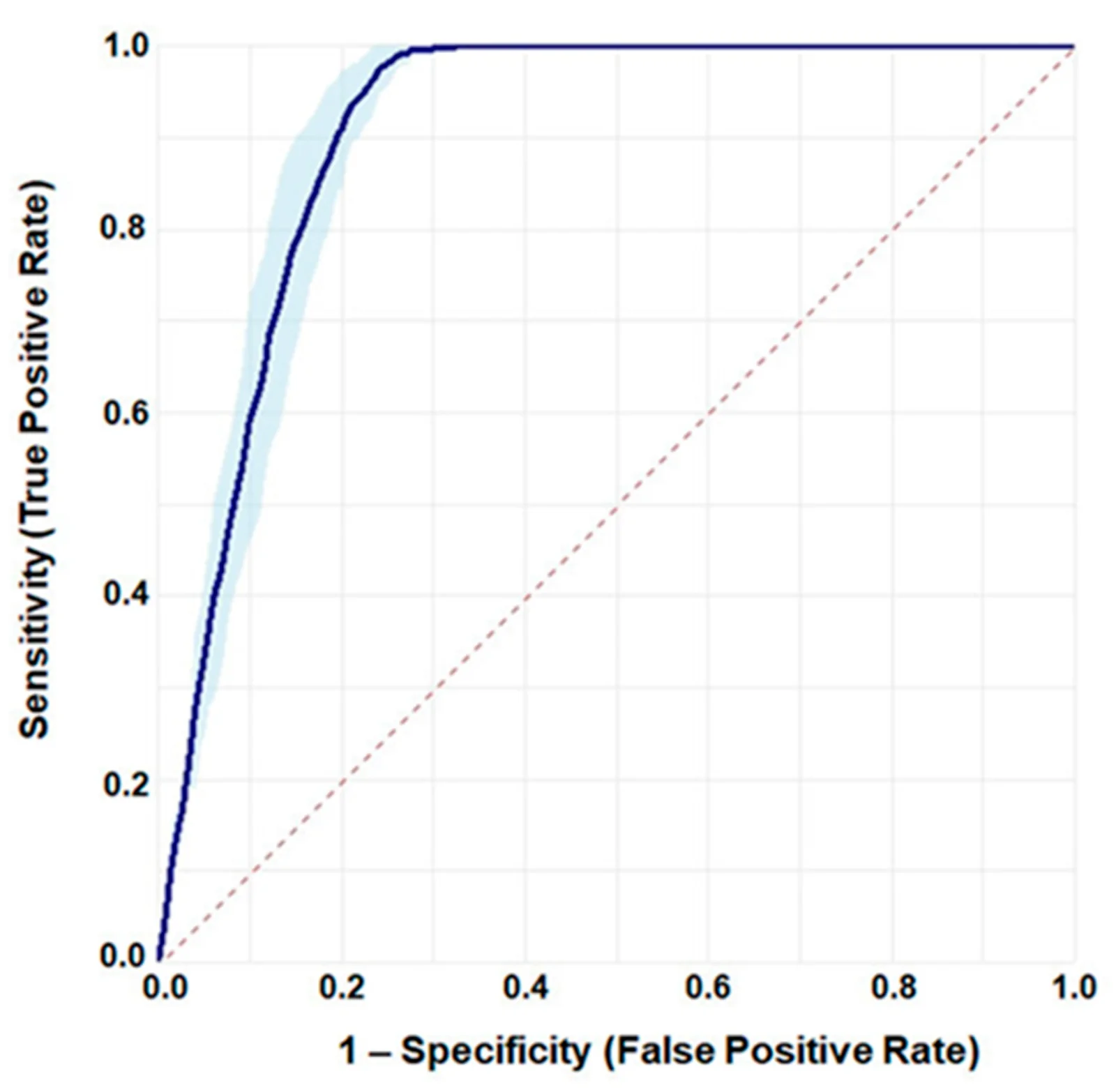
Mean receiver operating characteristic (ROC) curve for the MaxEnt model predicting *Apis dorsata* distribution. The shaded area represents the 95% confidence interval.

The optimized MaxEnt model used linear, quadratic, and hinge features (FC = LQH) with a regularization multiplier of 0.5. The model demonstrated good predictive performance, with a mean AUC of 0.905 ± 0.007 (Figure 2), indicating a high ability to discriminate between presence and background locations. The mean TSS was 0.746 ± 0.010, reflecting good agreement between observed and predicted distributions. The model also showed a low mean omission rate of 0.020 ± 0.002, indicating that only a small proportion of observed presence locations were incorrectly classified as unsuitable at the optimal threshold. At a mean optimal threshold of 0.236 ± 0.027, the model achieved a high mean sensitivity of 0.980 ± 0.002, indicating that most presence locations were correctly identified, while the mean specificity was 0.767 ± 0.009, demonstrating a moderate-to-high ability to distinguish unsuitable background locations. Model predictive performance was further supported by a mean CBI of 0.812 ± 0.062, indicating a generally high correspondence between predicted habitat suitability and the observed occurrence records.

### 3.2. Response Curves

**Figure 3:**
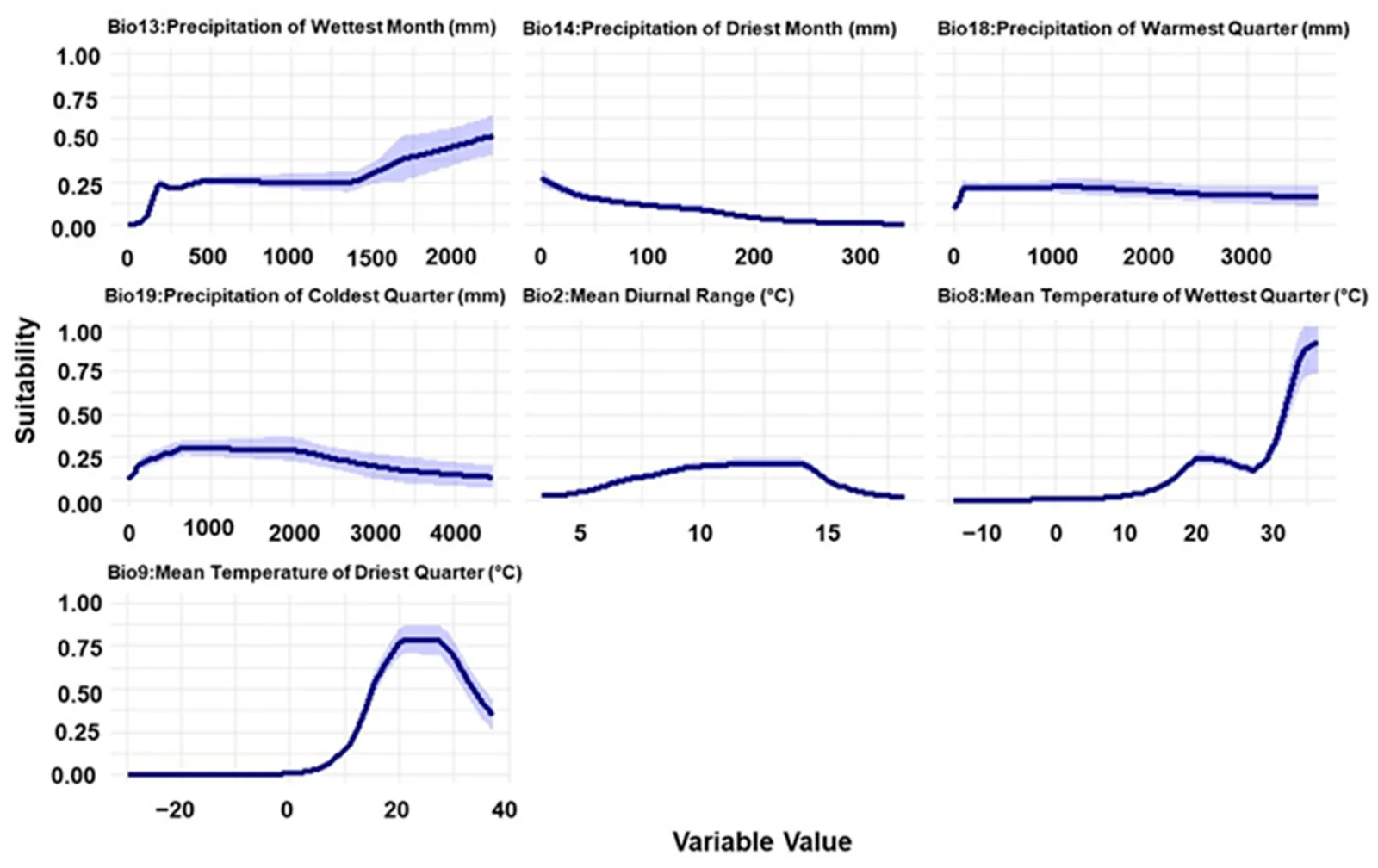
Response curves showing the relationship between the predicted habitat suitability of *Apis dorsata* and the bioclimatic variables used in the MaxEnt model. Solid lines represent the mean suitability response, and shaded bands represent ±1 SD across the five spatial cross-validation folds.

The response curves describing the relationship between predicted habitat suitability for *A. dorsata* and the seven bioclimatic variables included in the MaxEnt model are presented in Figure 3. Precipitation of the wettest month (bio13) showed an overall positive relationship with habitat suitability. Suitability increased rapidly from near zero at very low precipitation levels to approximately 0.20–0.25 at around 200–300 mm, followed by a relatively stable response of approximately 0.25–0.30 between about 400 and 1400 mm. At higher precipitation levels, suitability increased markedly, reaching approximately 0.60 at around 2200–2300 mm. This pattern suggests that *A. dorsata* is generally associated with moderate to high precipitation during the wettest month, with particularly high suitability under very wet conditions.

Precipitation of the driest month (bio14) exhibited a predominantly negative relationship with habitat suitability. Suitability was the highest at very low precipitation values, at approximately 0.30, and declined steadily as precipitation increased. Suitability approached near-zero values at approximately 250–330 mm, suggesting that relatively dry conditions during the driest month are more favorable for the species than high levels of precipitation.

Precipitation of the warmest quarter (bio18) showed a relatively stable response across most of its range. Suitability increased rapidly from approximately 0.10 at very low precipitation levels to around 0.20–0.23 at approximately 200–300 mm and remained relatively stable up to about 1200–1500 mm. Thereafter, suitability gradually declined, reaching approximately 0.15–0.18 at the highest precipitation values. This pattern indicates that low to moderate precipitation during the warmest quarter may provide relatively favorable conditions, whereas very high precipitation is associated with slightly lower suitability.

Precipitation of the coldest quarter (bio19) showed a moderate unimodal response. Suitability increased from approximately 0.10–0.15 at low precipitation levels to around 0.30 at approximately 500–800 mm and remained relatively stable across intermediate precipitation values. Suitability subsequently declined gradually as precipitation increased, reaching approximately 0.10–0.15 at around 4000–4500 mm. This suggests that intermediate levels of precipitation during the coldest quarter are more favorable than either very low or very high precipitation levels.

Mean diurnal range (bio2) displayed a unimodal relationship with habitat suitability. Suitability was low at values below approximately 5 °C, increased steadily to approximately 0.20–0.23 at around 9–13 °C, and remained relatively stable across this intermediate range. Suitability then declined sharply above approximately 14 °C and approached near-zero values at around 17–18 °C. This response indicates that *A. dorsata* is favored by moderate daily temperature fluctuations, whereas very low or particularly high diurnal temperature ranges are less suitable.

The mean temperature of the wettest quarter (bio8) showed one of the strongest and most complex responses among the variables. Habitat suitability remained close to zero at temperatures below approximately 10 °C and increased gradually between about 10 and 20 °C, reaching approximately 0.25–0.30 around 20–22 °C. Suitability then declined slightly at approximately 25–28 °C before increasing sharply above approximately 28–30 °C, reaching values close to 0.90 at around 34–35 °C. This pattern indicates a particularly strong association between *A. dorsata* and very warm conditions during the wettest quarter.

The mean temperature of the driest quarter (bio9) also exhibited a strong unimodal response. Suitability remained close to zero at temperatures below approximately 0 °C and increased gradually between approximately 0 and 15 °C. Suitability then rose sharply, reaching a maximum of approximately 0.80–0.85 at around 20–25 °C, where it remained relatively high over a narrow temperature range. Above approximately 25 °C, suitability declined progressively, reaching approximately 0.35–0.40 at around 38 °C. This response suggests that moderately warm temperatures during the driest quarter provide the most favorable conditions for *A. dorsata*, whereas both cooler and extremely warm conditions are associated with lower predicted suitability.

### 3.3. Climatic Determinants of Suitability

**Figure 4:**
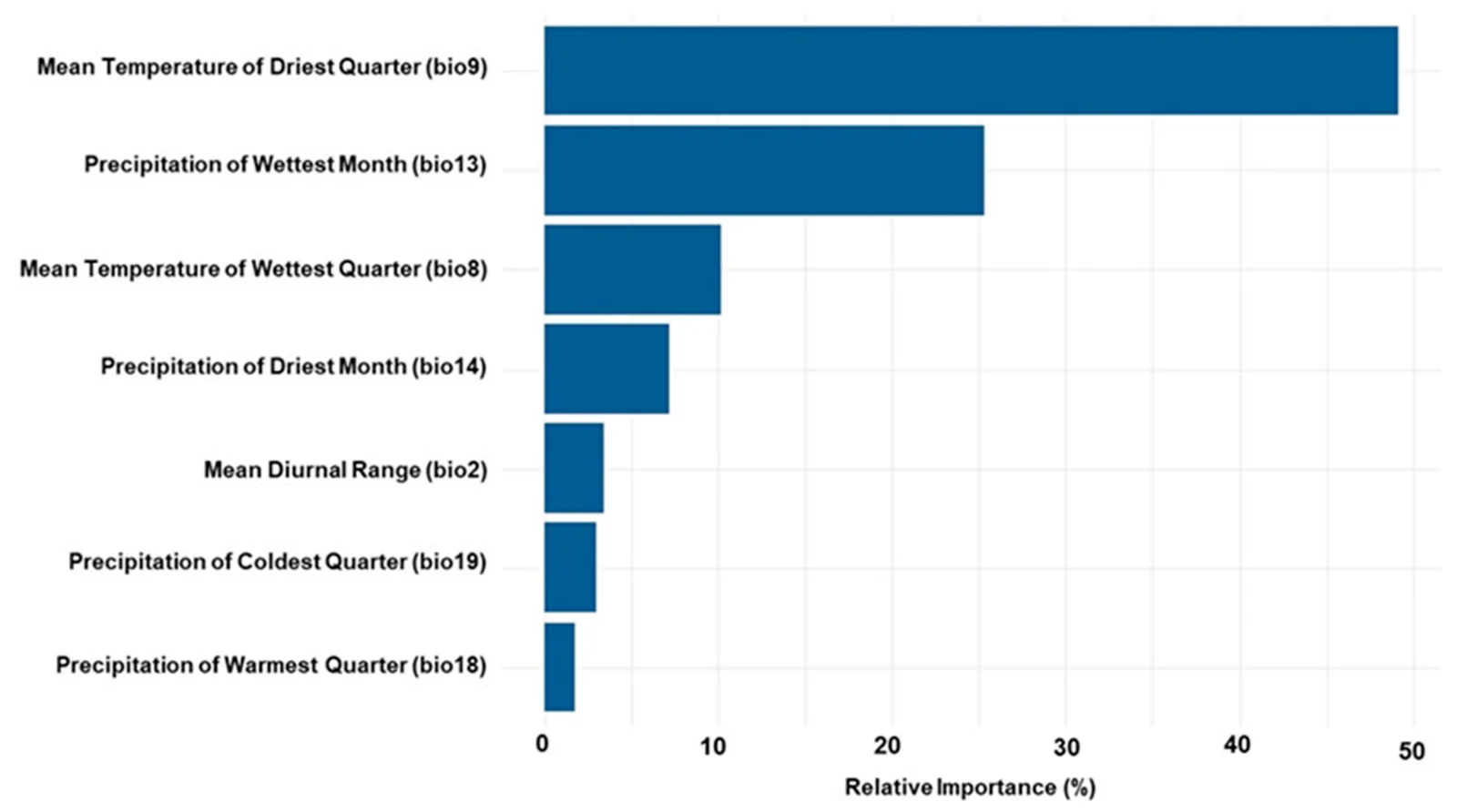
Relative importance (%) of bioclimatic variables used in the MaxEnt model.

Among the seven bioclimatic variables included in the optimized MaxEnt model (Figure 4), mean temperature of the driest quarter (bio9) was identified as the most influential predictor, with a relative importance of 49.17%, indicating that temperature conditions during the driest period play a dominant role in determining suitable habitat conditions for *A. dorsata*. Precipitation of the wettest month (bio13) ranked second, contributing 25.36%, highlighting the substantial importance of precipitation during the wettest period in shaping the species’ predicted distribution. Mean temperature of the wettest quarter (bio8) was the third most influential variable, with a relative importance of 10.20%, followed by precipitation of the driest month (bio14), which contributed 7.18%. Mean diurnal range (bio2) had comparatively lower importance, accounting for 3.44%. In contrast, precipitation of the coldest quarter (bio19) and precipitation of the warmest quarter (bio18) had relatively low importance, accounting for 2.95% and 1.70%, respectively.

### 3.4. Present Distribution of Habitat Suitability

**Figure 5:**
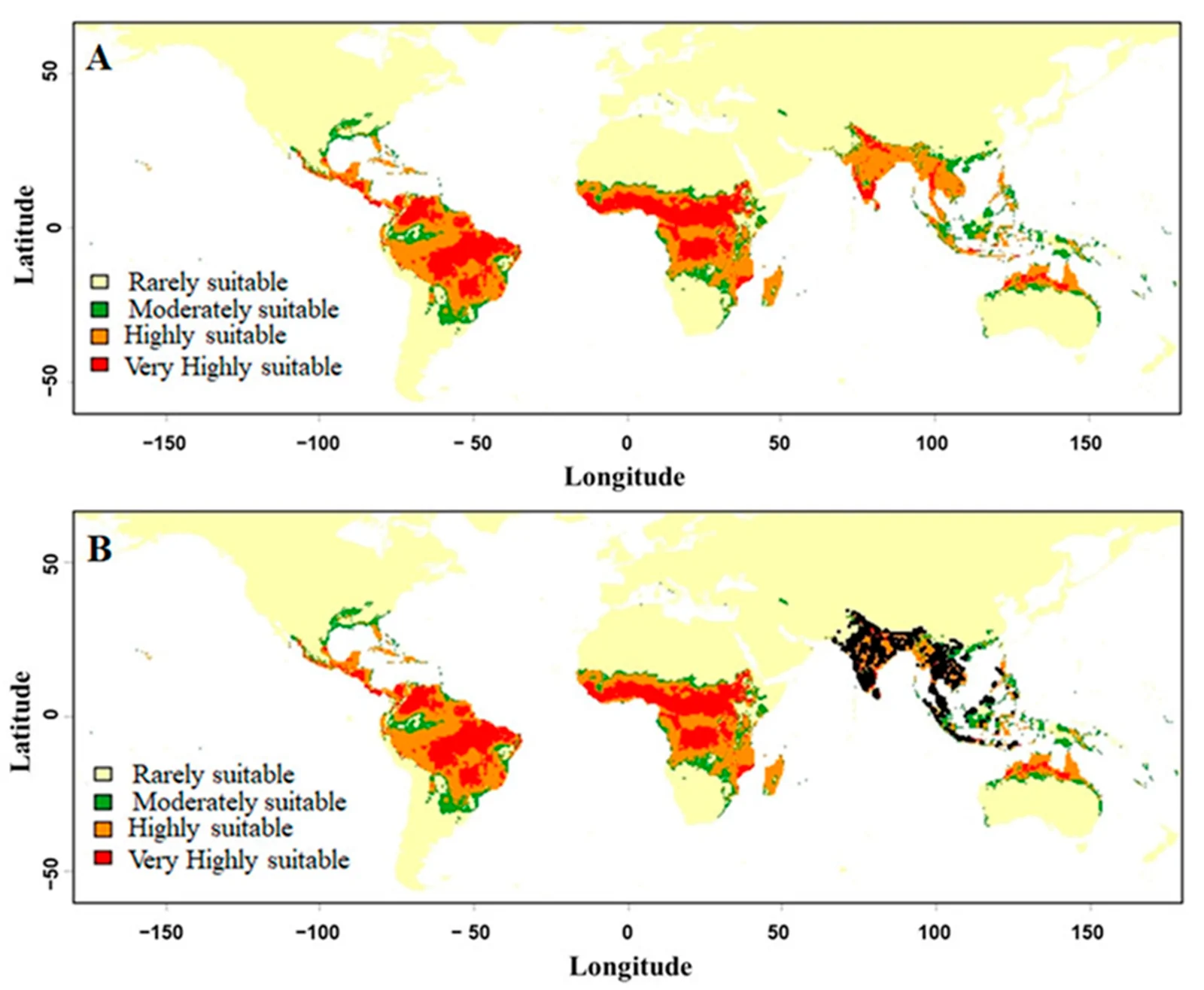
Current habitat suitability of *Apis dorsata* predicted using the MaxEnt model. (**A**) Map without occurrence records overlaid. (**B**) Map with occurrence records overlaid.

The global habitat suitability projection for *A. dorsata* is presented in Figure 5. The model predicted suitable habitats predominantly across tropical and subtropical regions, encompassing the species’ native range in South and Southeast Asia, as well as climatically suitable areas in Central and South America, tropical and southern Africa, and northern Australia. The suitable areas identified outside the native range should be interpreted as potential establishment or invasion-risk zones under scenarios involving the accidental or intentional transfer of *A. dorsata*, rather than as confirmed occurrences. Areas classified as very highly suitable were particularly concentrated in parts of South and Southeast Asia, with additional areas occurring in South America, Africa, and northern Australia. Highly suitable habitats were also broadly distributed across these tropical regions, while moderately suitable areas generally surrounded or occurred alongside the highly suitable zones. Rarely suitable habitats occupied much of the remaining projected global extent.

Based on the categorized global projection, rarely suitable habitats represented the largest proportion of the projected area, accounting for approximately 69.06%. Moderately suitable habitats accounted for approximately 7.09%, while highly suitable habitats represented approximately 14.87%. Very highly suitable habitats accounted for approximately 8.98% of the projected area. Overall, approximately 23.85% of the projected global area was classified as highly or very highly suitable, whereas approximately 76.15% fell within the rarely or moderately suitable categories. Figure 5B further shows the spatial distribution of the occurrence records in relation to the predicted habitat suitability patterns, providing a visual comparison between the observed records and the model’s projected suitable areas.

### 3.5. Projected Future Conditions for 2050

**Figure 6:**
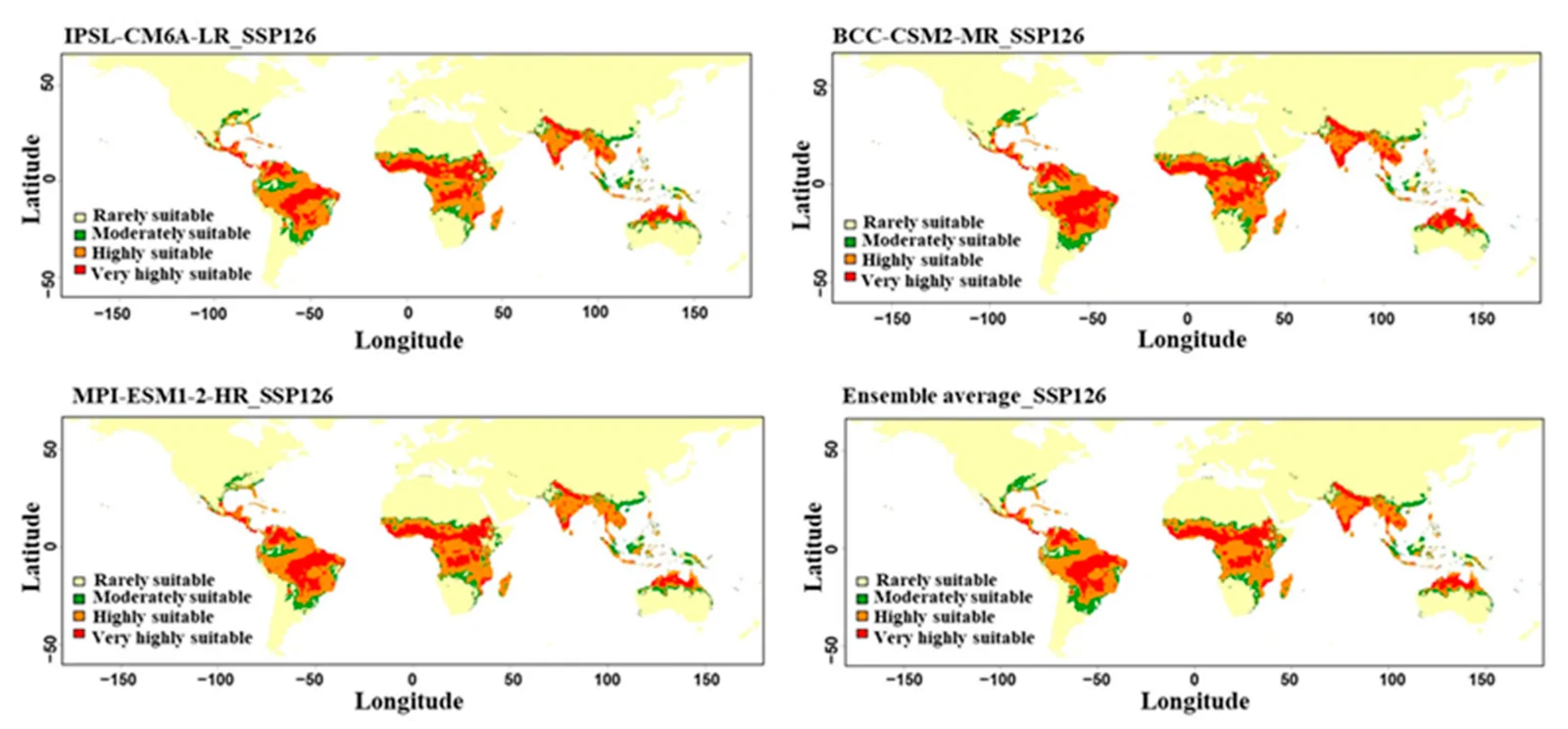
Predicted habitat suitability distribution of *Apis dorsata* for 2041–2060 (2050) under the SSP126 scenario based on three climate models (IPSL-CM6A-LR, BCC-CSM2-MR, and MPI-ESM1-2-HR), together with the ensemble average across the three models.

**Figure 7:**
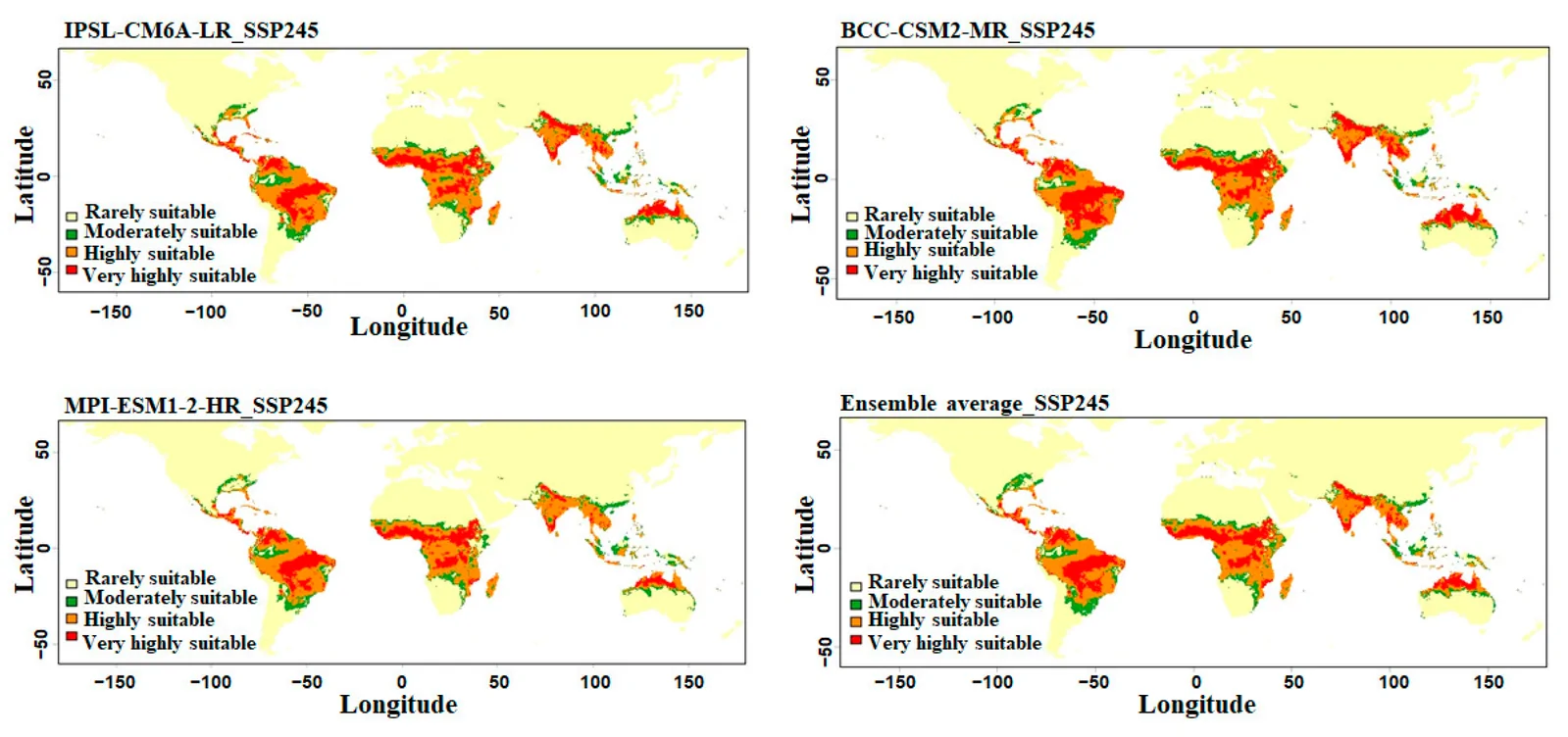
Predicted habitat suitability distribution of *Apis dorsata* for 2041–2060 (2050) under the SSP245 scenario based on three climate models (IPSL-CM6A-LR, BCC-CSM2-MR, and MPI-ESM1-2-HR), together with the ensemble average across the three models.

**Figure 8:**
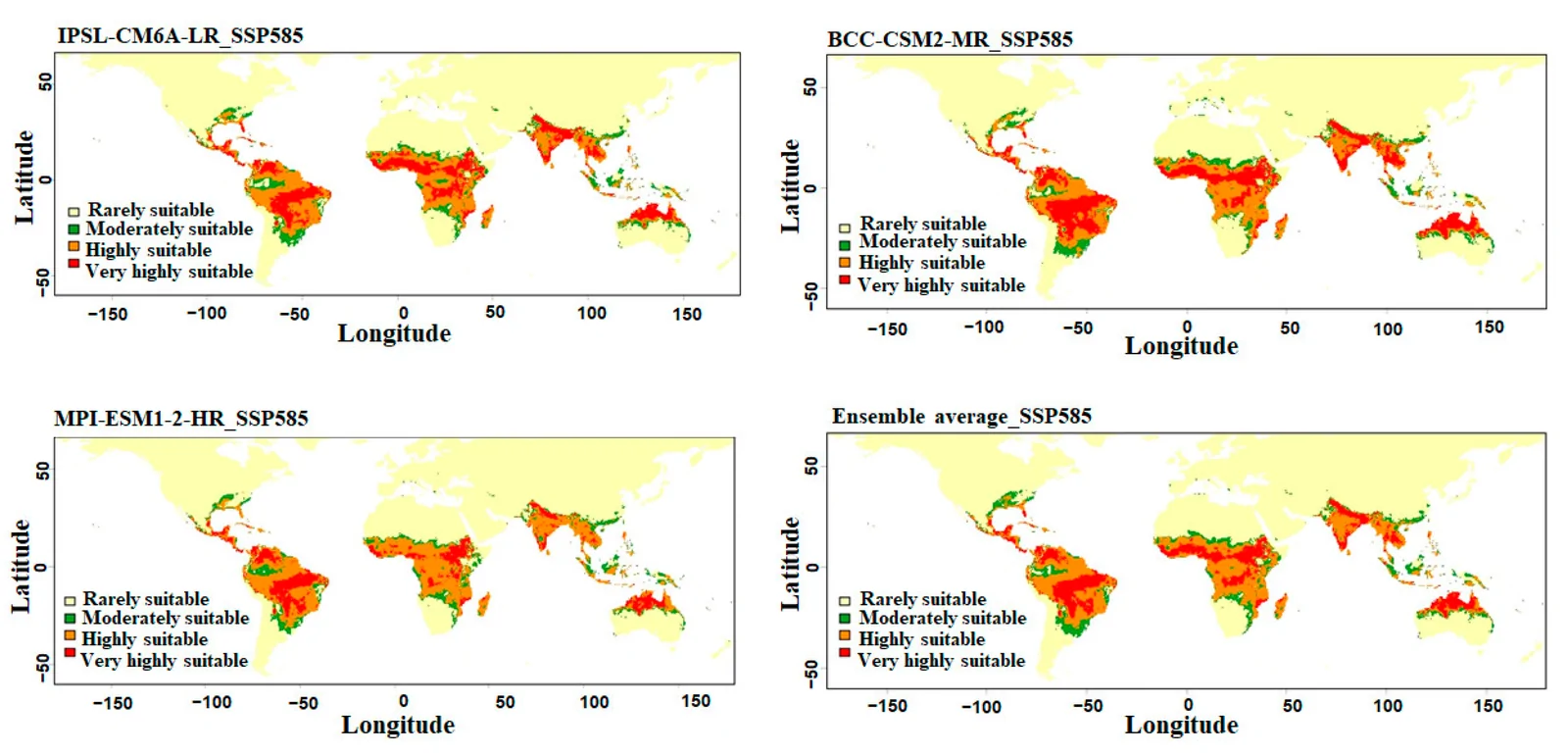
Predicted habitat suitability distribution of *Apis dorsata* for 2041–2060 (2050) under the SSP585 scenario based on three climate models (IPSL-CM6A-LR, BCC-CSM2-MR, and MPI-ESM1-2-HR), together with the ensemble average across the three models.

The projections for 2050 under SSP126, SSP245, and SSP585 (Figure 6, Figure 7 and Figure 8; Supplementary Table S2; File S4) showed broadly consistent spatial patterns across the three climate models (IPSL-CM6A-LR, BCC-CSM2-MR, and MPI-ESM1-2-HR), with the ensemble projections generally confirming the overall agreement. Suitable habitats were primarily distributed across the native range in South and Southeast Asia, in addition to other tropical and subtropical regions of Central and South America, Africa, and northern Australia, which, as noted in Section 3.4, should be considered potential establishment or invasion-risk zones rather than areas of confirmed occurrence. Highly and very highly suitable areas were particularly concentrated in South and Southeast Asia, with additional suitable areas occurring in parts of South America, Africa, and northern Australia.

Under SSP126 in 2050 (Figure 6), the three climate models showed substantial spatial agreement, although differences were evident in the relative proportions of suitability classes. BCC-CSM2-MR predicted the largest proportion of very highly suitable habitat (11.75%), whereas IPSL-CM6A-LR and MPI-ESM1-2-HR predicted 9.11% and 8.93%, respectively. The proportion of highly suitable habitat was relatively consistent among the models, ranging from 15.29% in BCC-CSM2-MR to 16.39% in IPSL-CM6A-LR. The proportion of moderately suitable habitat ranged from 6.34% in BCC-CSM2-MR to 7.15% in IPSL-CM6A-LR, while MPI-ESM1-2-HR predicted 6.84%. Rarely suitable habitat remained the dominant category across all models, accounting for approximately 66.62–68.24% of the projected area.

The ensemble-average projection for SSP126 indicated that rarely suitable habitat represented the largest proportion of the projected area (67.01%), followed by highly suitable habitat (16.17%), very highly suitable habitat (9.58%), and moderately suitable habitat (7.24%). Overall, highly and very highly suitable areas together represented approximately 25.75% of the projected area, whereas rarely and moderately suitable habitats accounted for approximately 74.25%.

Under SSP245 (Figure 7), the projections from the three climate models showed broadly similar spatial patterns, although differences were evident in the relative proportions of the habitat suitability classes. The IPSL-CM6A-LR projection classified approximately 66.94% of the projected area as rarely suitable, 7.32% as moderately suitable, 16.34% as highly suitable, and 9.40% as very highly suitable. The BCC-CSM2-MR projection showed a slightly lower proportion of rarely suitable habitat (66.13%) and moderately suitable habitat (6.23%), while predicting 15.59% highly suitable and the largest proportion of very highly suitable habitat (12.05%) among the three models. In contrast, the MPI-ESM1-2-HR projection indicated the largest proportion of rarely suitable habitat (68.21%) and predicted 6.95% moderately suitable habitat, 16.74% highly suitable habitat, and 8.10% very highly suitable habitat.

The ensemble-average projection provided an intermediate representation of the three climate-model outputs, with approximately 66.61% rarely suitable, 7.33% moderately suitable, 16.62% highly suitable, and 9.44% very highly suitable habitat. Overall, highly and very highly suitable habitats together represented approximately 26.05% of the projected area under SSP245, whereas rarely and moderately suitable habitats accounted for approximately 73.95%.

Compared with the ensemble projection under SSP126, SSP245 resulted in a slight increase in the proportion of highly suitable habitat, from 16.17% to 16.62%, while very highly suitable habitat showed a slight decrease from 9.58% to 9.44%. Consequently, the combined proportion of highly and very highly suitable habitats increased marginally from 25.75% under SSP126 to 26.06% under SSP245. At the same time, the proportion of rarely suitable habitat declined slightly from 67.01% to 66.61%, indicating relatively minor changes in the overall extent and spatial distribution of suitable habitat for *A. dorsata* under the SSP245 scenario.

Under SSP585 (Figure 8), the projections from the three climate models showed broadly similar spatial patterns, although differences were evident in the proportions of the habitat suitability classes. The proportion of rarely suitable habitat ranged from approximately 65.35% in BCC-CSM2-MR to 67.23% in MPI-ESM1-2-HR, with IPSL-CM6A-LR predicting approximately 66.09%. Thus, despite the higher-emission SSP585 scenario, the majority of the projected area remained within the lowest suitability class. The IPSL-CM6A-LR projection classified approximately 7.06% of the projected area as moderately suitable, 16.31% as highly suitable, and 10.54% as very highly suitable. The BCC-CSM2-MR model predicted 6.45% moderately suitable and 15.79% highly suitable habitat, while producing the largest proportion of very highly suitable habitat (12.41%) among the three models. In contrast, the MPI-ESM1-2-HR projection indicated 6.84% moderately suitable habitat and the largest proportion of highly suitable habitat (18.38%), but the smallest proportion of very highly suitable habitat (7.55%).

The ensemble projection showed that rarely suitable habitats accounted for approximately 65.86% of the projected area, representing the largest suitability category. Moderately suitable habitats accounted for approximately 7.14%, while highly suitable habitats represented 16.98% and very highly suitable habitats accounted for 10.02%. Consequently, the combined proportion classified as highly or very highly suitable was approximately 27.00%, whereas 73.00% was classified as rarely or moderately suitable.

Compared with the lower-emission scenarios, the ensemble projections indicate a gradual increase in the proportion of highly suitable habitat, from 16.17% under SSP126 to 16.62% under SSP245 and 16.98% under SSP585. However, the combined proportion of highly and very highly suitable habitat changed only modestly, increasing from 25.75% under SSP126 to 26.06% under SSP245 and 27.00% under SSP585. At the same time, the proportion of rarely suitable habitat declined from 67.01% under SSP126 to 66.61% under SSP245 and 65.86% under SSP585. Overall, the SSP585 projections suggest a modest expansion of highly and very highly suitable habitats for *A. dorsata*, particularly across tropical and subtropical regions, although the spatial patterns and magnitude of change varied among the three climate models.

The grand overall ensemble projection for 2050, averaged across the three climate models and three emissions scenarios (Supplementary Figure S4; File S3), showed that environmentally suitable areas for *A. dorsata* were primarily concentrated in tropical and subtropical regions, particularly in South and Southeast Asia, Central and South America, sub-Saharan Africa, and northern Australia. Rarely suitable habitats represented the largest proportion of the projected global area, accounting for 66.45%, followed by highly suitable habitats (16.61%), very highly suitable habitats (9.67%), and moderately suitable habitats (7.27%). Overall, the combined proportion of highly and very highly suitable habitats was 26.28%, whereas 73.72% of the projected area was classified as rarely or moderately suitable. These results indicate that, despite variation among climate models and emissions scenarios, substantial areas of potentially suitable habitat for *A. dorsata* are expected to persist across tropical and subtropical regions by 2050.

### 3.6. Geographic Range Shift Analysis 

**Figure 9:**
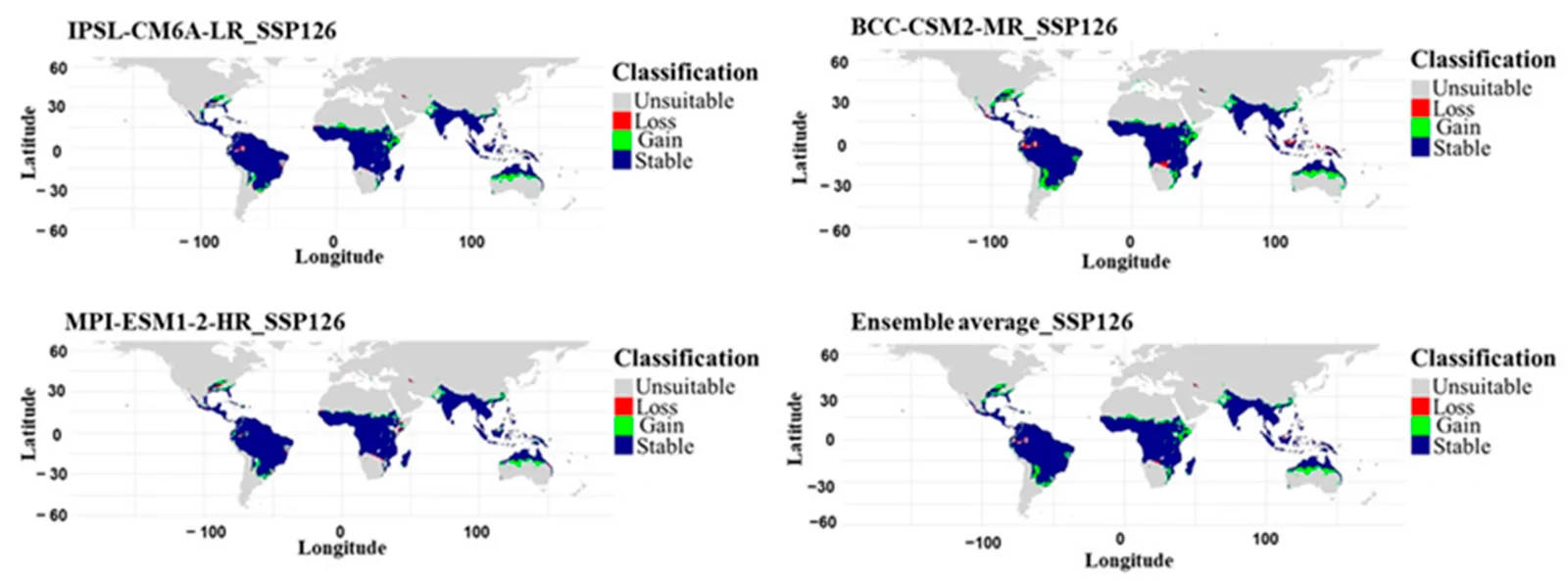
Projected habitat suitability dynamics of *Apis dorsata* between current conditions and 2050 under the SSP126 scenario based on three climate models (IPSL-CM6A-LR, BCC-CSM2-MR, and MPI-ESM1-2-HR), along with the ensemble average across the three models.

**Figure 10:**
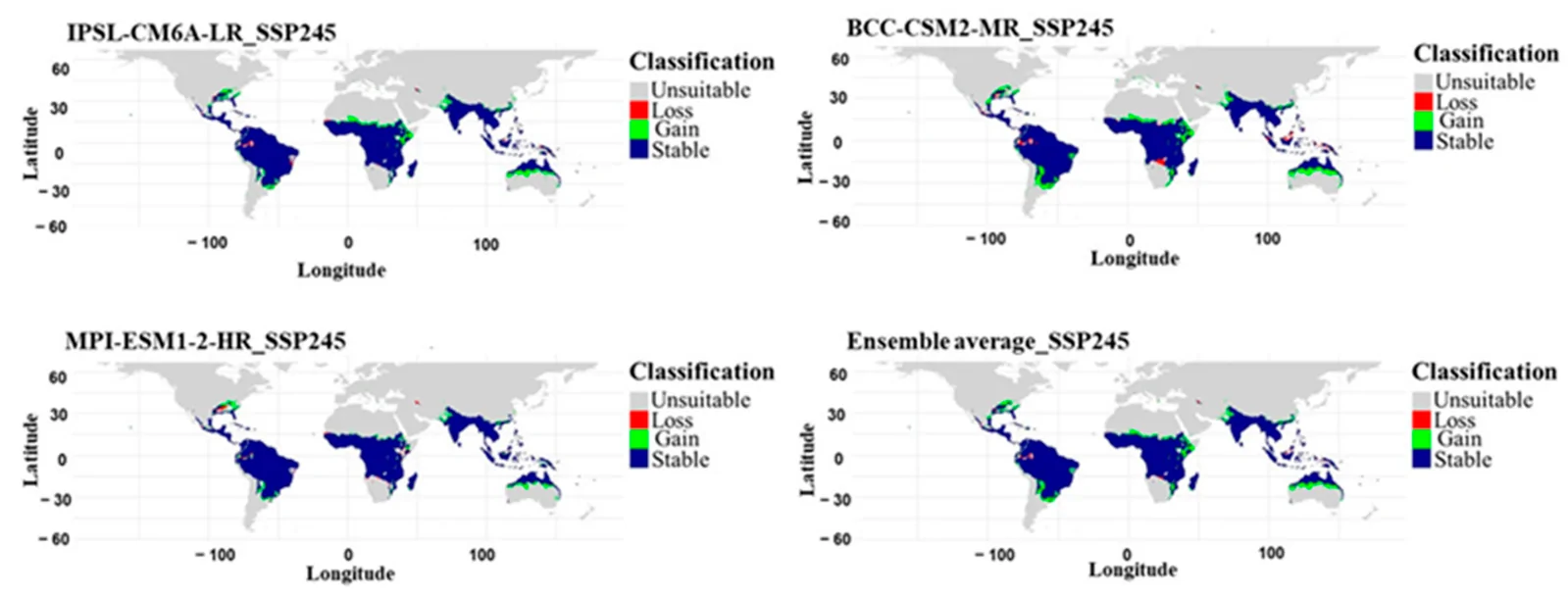
Projected habitat suitability dynamics of *Apis dorsata* between current conditions and 2050 under the SSP245 scenario based on three climate models (IPSL-CM6A-LR, BCC-CSM2-MR, and MPI-ESM1-2-HR), along with the ensemble average across the three models.

**Figure 11:**
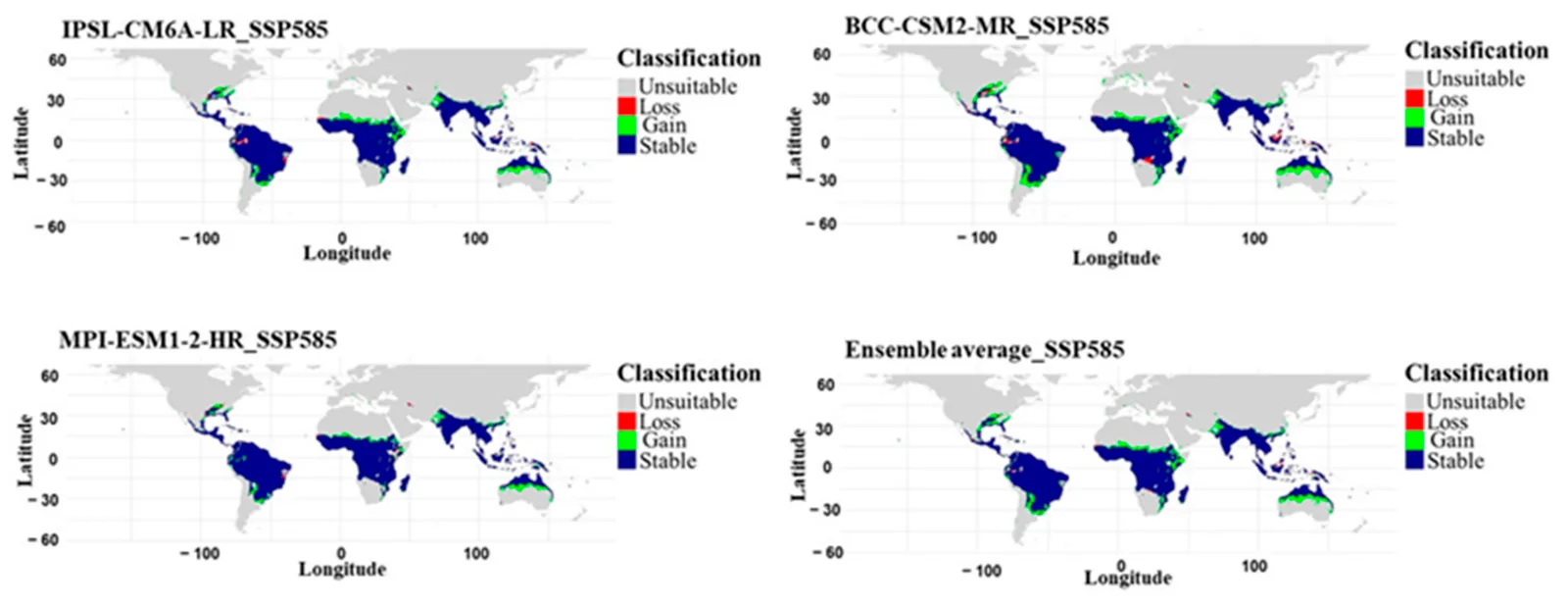
Projected habitat suitability dynamics of *Apis dorsata* between current conditions and 2050 under the SSP585 scenario based on three climate models (IPSL-CM6A-LR, BCC-CSM2-MR, and MPI-ESM1-2-HR), along with the ensemble average across the three models.

The projected changes in habitat suitability for *A. dorsata* between current conditions and 2050 under SSP126, SSP245, and SSP585 are presented in Figure 9, Figure 10 and Figure 11 and Supplementary Table S3, File S4. Across the three climate models, the projected spatial patterns were broadly consistent, with a substantial proportion of suitable habitat remaining stable and projected habitat gains generally exceeding habitat losses. Gains were primarily concentrated within and around existing suitable areas across tropical and subtropical regions, particularly in Central and South America, Africa, and South and Southeast Asia, whereas habitat losses were relatively limited and spatially fragmented.

Under SSP126 (Figure 9), the IPSL-CM6A-LR model projected 30.27% of the global area as stable habitat, 2.75% as habitat gain, and 0.34% as habitat loss. The corresponding values for BCC-CSM2-MR were 29.82% stable habitat, 3.90% habitat gain, and 0.80% habitat loss, respectively. MPI-ESM1-2-HR projected 30.28% stable habitat, 1.86% habitat gain, and 0.34% habitat loss. The SSP126 ensemble projection indicated that 30.32% of the global area would remain stable, while 3.03% was projected to gain suitability and only 0.30% to experience habitat loss. Overall, these results indicate that under the SSP126 scenario, most currently suitable areas for *A. dorsata* are expected to remain stable by 2050, with projected habitat gains exceeding losses across all three climate models.

Under SSP245 (Figure 10), the projected spatial shift patterns were broadly consistent across the three climate models, with stable habitat representing the largest proportion of the projected area and habitat gains generally exceeding habitat losses. The spatial distribution shown indicates that stable habitats were concentrated across the major currently suitable regions, while gains occurred primarily around the margins of these areas and losses were relatively limited and fragmented.

For IPSL-CM6A-LR, stable habitat accounted for 30.24% of the projected area, while habitat gain and loss accounted for 3.20% and 0.38%, respectively. The BCC-CSM2-MR model projected 29.78% stable habitat and showed the greatest habitat gain among the three models (4.43%), while habitat loss accounted for 0.84%. In contrast, MPI-ESM1-2-HR projected the largest proportion of stable habitat (30.28%) among the three models, with habitat gain accounting for 1.86% and habitat loss for 0.33%.

The SSP245 ensemble projection showed that 30.30% of the projected area would remain stable, while 3.43% was projected to experience habitat gain and only 0.32% was projected to experience habitat loss.

Under SSP585 (Figure 11), the projected spatial shift patterns remained broadly consistent across the three climate models, with stable habitat accounting for the largest proportion of the projected area and habitat gains exceeding habitat losses. Stable habitats remained concentrated across the major suitable regions, while projected gains were generally distributed along the margins of existing suitable areas. Habitat losses were comparatively limited and spatially fragmented.

For IPSL-CM6A-LR, stable habitat accounted for 30.18% of the projected area, while habitat gain and loss accounted for 4.10% and 0.43%, respectively. The BCC-CSM2-MR model projected 29.82% stable habitat and the greatest habitat gain among the three models (5.19%), while habitat loss accounted for 0.80%. In contrast, MPI-ESM1-2-HR projected the largest proportion of stable habitat (30.31%), with habitat gain accounting for 2.76% and habitat loss for 0.30%. The SSP585 ensemble projection indicated that 30.36% of the projected area would remain stable, while 4.15% was projected to gain suitability and only 0.26% was projected to experience habitat loss.

Overall, unsuitable areas constituted the largest proportion of the global study area under all scenarios, although their proportion declined progressively from 66.35% under SSP126 to 65.95% under SSP245 and 65.23% under SSP585 in the ensemble projections. Consistent with this pattern, projected habitat gains increased from 3.03% under SSP126 to 3.43% under SSP245 and 4.15% under SSP585. Habitat losses remained consistently low, accounting for 0.30%, 0.32%, and 0.26%, respectively, while stable habitat remained relatively constant at approximately 30.3% across all scenarios. These results indicate that projected habitat expansion consistently exceeds habitat contraction under future climate scenarios, with the greatest expansion projected under SSP585.

The projected global range shifts of *A. dorsata* by 2050, comparing current habitat suitability with the grand overall future ensemble averaged across the three climate models and three emission scenarios (Supplementary Figure S5; File S3), indicate that most currently suitable areas are projected to remain stable. Stable habitats were particularly concentrated across South and Southeast Asia, tropical Africa, Central and South America, and parts of Oceania, while habitat gains occurred mainly along the margins of existing suitable areas. Habitat losses were comparatively limited and spatially fragmented.

Overall, the grand ensemble classified 30.35% of the global area as stable habitat, 3.55% as habitat gain, and only 0.26% as habitat loss, while 65.84% remained unsuitable. The substantially greater proportion of habitat gain relative to habitat loss indicates a net expansion of approximately 3.29% in climatically suitable habitat.

## 4. Discussion

The optimized MaxEnt model demonstrated high predictive performance and good discrimination, indicating that the selected environmental predictors and model configuration captured important aspects of the ecological niche of *A. dorsata*. The use of linear, quadratic, and hinge features with an appropriately optimized level of regularization appears to have provided sufficient model flexibility to represent potentially complex species–environment relationships while limiting overfitting. The consistently good performance across spatial cross-validation folds further supports the robustness and transferability of the model. These findings are consistent with previous studies showing that MaxEnt can provide reliable species-distribution predictions when appropriate environmental variables, feature classes, regularization settings, and validation procedures are applied [25,26,27].

The low omission rate and high sensitivity indicate that the model was able to identify environmentally suitable conditions associated with known occurrence locations. This is especially important for applications in which failing to identify potentially suitable habitats could limit conservation planning, ecological assessments, or future distribution analyses. However, the comparatively lower specificity indicates that some background locations shared environmental characteristics with known occurrence sites and were consequently predicted as potentially suitable. Such patterns do not necessarily indicate poor model performance because environmentally suitable areas may remain unoccupied due to factors not fully represented by the environmental predictors, including dispersal limitations, biotic interactions, habitat accessibility, and human disturbance [18,28]. Therefore, the predicted distribution should be interpreted primarily as an estimate of potential environmental suitability rather than confirmed species occupancy, particularly in areas where favorable environmental conditions may occur outside the realized distribution of *A. dorsata*. The high CBI further supports the model’s predictive performance, suggesting that locations assigned higher suitability values generally showed better correspondence with the observed occurrence pattern. This provides additional evidence that the model was not only able to discriminate between suitable and less suitable environmental conditions but also to rank predicted suitability in a meaningful manner. Similar interpretations of high CBI values have been reported as evidence of good model calibration and ecologically informative predictions [25,28].

The response curves and relative importance of climatic predictors indicate that the potential distribution of *A. dorsata* is shaped by the combined effects of seasonal temperature, precipitation, and daily thermal variability rather than by any single climatic factor. This pattern reflects the climate-dependent ecology of this tropical and subtropical honeybee [29,30]. However, the predictors differed in both their relative contributions and the nature of their relationships with habitat suitability. The response curves should be interpreted as correlative associations between environmental conditions and predicted suitability, conditional on the background data and the other predictors included in the model. Mean temperature during the driest quarter was the most influential predictor, indicating that thermal conditions during this potentially stressful period play a central role in shaping the climatic niche of *A. dorsata*. The response curve showed an association with moderately warm conditions, whereas both low and excessively high temperatures were less favorable. Suitable dry-season temperatures may support colony activity and resource use, while thermal extremes could impose physiological stress or coincide with reduced floral resources [5,30].

Precipitation during the wettest month was the second most influential predictor, highlighting the importance of moisture availability during the wet season. Suitability increased under high rainfall conditions, suggesting that substantial precipitation may enhance vegetation productivity, flowering, and the availability of nectar and pollen [3,31]. However, the effects of high rainfall may depend on local habitat characteristics and flowering phenology. Mean temperature during the wettest quarter also made a substantial contribution, reinforcing the importance of seasonal thermal conditions across contrasting periods of the year. Suitability increased substantially under very warm and wet conditions, potentially reflecting favorable interactions between temperature, moisture availability, and seasonal resource productivity. Together, the contributions of temperature during both the driest and wettest quarters suggest that the climatic niche of *A. dorsata* is shaped by seasonal thermal dynamics rather than average annual temperature alone [29,30].

Precipitation during the driest month made a moderate contribution. The response curve indicated greater suitability under relatively dry conditions, whereas high rainfall was less favorable. This suggests that seasonal dryness is an important component of the species’ climatic niche. *A. dorsata* may tolerate relatively dry conditions when suitable temperatures and floral resources are available, while its mobility and ability to exploit seasonal resources may reduce the effects of short-term reductions in rainfall [30,32]. Mean diurnal temperature range made a smaller but ecologically meaningful contribution. The response curve suggested that intermediate daily temperature fluctuations were more favorable, whereas large day–night differences reduced suitability. This pattern indicates that thermal variability provides ecological information beyond seasonal mean temperatures and may influence physiological demands and colony thermoregulation. Precipitation during the warmest and coldest quarters contributed relatively little compared with the other predictors, although their response curves revealed distinct patterns. Precipitation during the warmest quarter remained relatively stable across much of its range but declined under very high rainfall, suggesting a broad tolerance to moisture conditions during warm periods. In contrast, precipitation during the coldest quarter showed an intermediate optimum, with both low and high rainfall associated with reduced suitability. These lower contributions may indicate overlap with more influential seasonal predictors rather than ecological irrelevance [3,31,32].

The global habitat suitability projection indicates that the potential climatic distribution of *A. dorsata* is concentrated predominantly in tropical and subtropical regions. This pattern is consistent with the species’ association with warm environments and seasonal moisture availability [2,30]. The highest suitability was observed primarily in South and Southeast Asia, broadly corresponding to the recognized native distribution of *A. dorsata*. These regions may support colony maintenance and development by providing suitable food resources and environmental conditions [3,33]. Beyond the recognized native range, the model identified highly and very highly suitable conditions in parts of South America, Africa, Central America, and northern Australia. These projections represent climatically analogous environments rather than confirmed populations. The realized distribution may be constrained by land use, habitat accessibility, nesting sites, floral resources, biotic interactions, pathogens, predators, dispersal barriers, and human disturbance. These areas should therefore be interpreted as potential climatic space rather than confirmed habitat.

Highly favorable climatic conditions occupied a more restricted portion of the global landscape than low or marginal suitability areas. Moderately suitable areas generally occurred around highly suitable zones, suggesting transitional environments where some climatic requirements are met but the consistent combination of temperature and moisture associated with optimal habitats may be lacking. The predominance of rarely suitable areas emphasizes that favorable climatic conditions for *A. dorsata* are not uniformly distributed globally. Colder, arid, and highly seasonal environments may constrain foraging, vegetation productivity, floral-resource availability, and colony performance, thereby limiting the species’ broad potential range. The association of occurrence records with suitable regions provides an indication of model plausibility, whereas suitable areas without records may represent climatically favorable but unsampled, inaccessible, or unoccupied locations. This distinction is important because species-distribution models estimate environmental suitability rather than direct evidence of presence or absence [18,19].

The 2050 projections indicate that the potential climatic niche of *A. dorsata* is likely to remain concentrated predominantly within tropical and subtropical regions under all examined emissions scenarios. South and Southeast Asia remain the principal centers of high climatic suitability, with additional suitable areas persisting across parts of Central and South America, sub-Saharan Africa, and northern Australia. This overall persistence suggests that climate change over the modeled period is unlikely to fundamentally alter the species’ broad global distribution pattern, although localized changes in the extent and intensity of suitable conditions may occur. The broad agreement among the three climate models strengthens confidence in this general spatial pattern. The ensemble projections therefore provide a useful representation of the common climatic signal while accounting for variation among individual climate-model outputs. Across the emissions scenarios, projected changes in overall suitability were relatively gradual. The combined extent of highly and very highly suitable conditions changed only modestly among scenarios, indicating localized expansion or intensification of favorable conditions rather than a major global reorganization of the species’ potential distribution by 2050. Large areas also remained within the lowest suitability category, particularly in temperate, high-latitude, and highly arid regions, indicating that warming alone may not overcome fundamental climatic constraints related to seasonal temperature and moisture regimes.

Some areas outside the recognized distribution continued to show suitable climatic conditions under future scenarios, suggesting that climate change may maintain or increase the availability of potentially favorable environments. Nevertheless, climatic suitability alone does not demonstrate that the species will disperse naturally or establish persistent populations in these areas. Future projections outside the recognized range should therefore be interpreted as representing potential climatic opportunity rather than evidence of inevitable range expansion. Newly suitable areas adjacent to existing populations or connected by favorable landscapes may be more accessible than isolated patches separated by unsuitable environments or geographic barriers. The high mobility of *A. dorsata* and its capacity to track seasonal floral resources may facilitate the use of newly favorable environments [30,32]. However, barriers, habitat fragmentation, and limited floral resources may still restrict movement and establishment.

Human-mediated transport can overcome natural dispersal barriers and has already contributed to the introduction of other *Apis* species into new regions [34,35,36,37]. The successful interception of *A. florea* at a U.S. shipping port illustrates the importance of rapid detection and containment in preventing establishment following introduction [38]. Similarly, the detection and containment of *A. dorsata* on a cargo vessel at the Port of Elizabeth, New Jersey, in 2025 demonstrates that human transport can provide a pathway through which the species may reach areas beyond its established range [39]. Accordingly, areas that remain or become climatically suitable outside the recognized distribution may support temporary survival or establishment following accidental introduction if other ecological requirements are met. The persistence of broad climatic suitability should also not be interpreted as evidence that existing *A. dorsata* populations will remain unaffected by climate change. Future assessments would therefore benefit from integrating land cover, vegetation productivity, floral phenology, nesting resources, and other ecological variables with climatic predictors [30,32].

The projected habitat dynamics suggest that habitat gains potentially exceed losses and that much of the currently suitable climatic space remains stable. Future climate change may therefore create additional favorable conditions in some regions while causing limited contraction in others. However, these projections represent changes in potential climatic suitability rather than direct changes in the realized distribution of the species. Moderate warming may shift some currently marginal environments into a more favorable climatic range. The projected increase in habitat gains under stronger climate forcing is broadly consistent with the modeled responses of *A. dorsata* to seasonal temperature conditions. Similarly, stronger warming scenarios suggest potential expansion beyond the species’ current range [40]. However, habitat gains represent expanded climatic opportunity rather than a direct prediction of successful colonization or establishment. Moreover, these projected gains may not translate into viable or persistent *A. dorsata* populations, as stronger warming could introduce ecological constraints through excessive heat, drought, altered precipitation, seasonal shifts, and changes in floral resource availability. Although projected habitat loss may be less extensive than habitat gain globally, localized contractions could have important ecological consequences. Losses affecting major populations, nesting habitats, or corridors connecting suitable regions may disproportionately influence population persistence and connectivity [2,41]. Regional analyses are therefore needed to determine whether projected losses overlap with ecologically important areas. 

While macroecological assessments often project overall global habitat stability or broad-scale expansion, these global metrics can obscure severe localized vulnerabilities. For instance, regional modeling indicates that specialized or range-edge environments, such as the vulnerable arid and alpine zones of Pakistan, may experience substantial contractions under severe warming scenarios [2]. Consequently, broad global stability does not preclude severe regional declines, emphasizing the need for localized assessments to capture the fine-scale pressures faced by regional populations. To inform long-term habitat management, these dynamic projections highlight the dual need to monitor potential expansion corridors while maintaining habitat quality within persistent core ranges. Newly suitable areas may become future colonization zones, especially where they are connected to existing populations and contain appropriate vegetation and nesting resources. At the same time, maintaining habitat quality within persistent core areas remains essential because climatic stability does not protect populations from local habitat degradation and other non-climatic pressures.

## 5. Conclusions

Using an optimized MaxEnt ecological niche modeling framework, this study demonstrated that the current and future potential distribution of *A. dorsata* is primarily governed by the combined effects of seasonal temperature and moisture availability. Good model performance indicated high correspondence between predicted suitability and the species’ recognized distribution in tropical and subtropical Asia, while projections also identified climatically suitable areas in non-native regions, including Africa, the Americas, and northern Australia. Under 2050 climate scenarios, suitable climatic conditions are projected to persist and, in some areas, expand, with projected habitat gains generally exceeding losses. These projections are conditional on the assumption that the present-day climatic niche of *A. dorsata* remains stable under future conditions, an inherent assumption of correlative SDMs that may become increasingly uncertain when projecting into novel climatic environments. However, these shifts should be interpreted as changes in potential environmental suitability rather than evidence of guaranteed dispersal, colonization, or establishment. The realized distribution remains constrained by other factors, including dispersal limitations, habitat accessibility, floral-resource availability, nesting-site availability, land-use change, and biotic interactions. From a management perspective, these findings support targeted microclimate and habitat monitoring within the native range, as well as enhanced surveillance at major ports and other potential entry points in highly suitable regions outside the native range. Such measures can strengthen pollinator conservation, ecological monitoring, and biosecurity planning under future climate change.

## Data Availability

The data supporting the findings of this study are included within the article and its Supplementary Materials. Additional data are available from the corresponding author upon reasonable request.

## Supplementary Materials

The following supporting information can be downloaded at https://www.mdpi.com/article/10.3390/insects17090954/s1. File S1: Dataset of 1060 records used in the study. File S2: R code. File S3: Supplementary Figures. File S4: Supplementary Tables.

## Abbreviations

The following abbreviations are used in this manuscript:

MaxEnt	Maximum Entropy
SSP	Shared Socioeconomic Pathway
AUC	Area Under the Curve
TSS	True Skill Statistic
