# Supplementary material for: Global Habitat Suitability Modeling of the Giant Honeybee (Apis dorsata) Under Future Climate Change Scenarios
Source: Insects. 2026 Sep 12;17(9):954. doi: 10.3390/insects17090954 (PMC13607631; doi:10.3390/insects17090954)
Supplement: Supplementary file 1 [file insects-17-00954-s001.zip › File S2.pdf]

Examples of the R code used in the methodology section

```r
library(tidyverse)
library(sf)
library(CoordinateCleaner)
library(spThin)
library(skimr)
library(corrplot)
library(rnaturalearth)
library(rnaturalearthdata)
library(ggspatial)
library(terra)
library(usdm)
library(virtualspecies)
library(maxnet)
library(dismo)
library(ggplot2)
library(pROC)
library(ecospat)
library(ENMeval)
library(blockCV)
library(rnaturalearth)


input_file   <- "C:/"
output_file  <- "C:/"
summary_file <- sub("\\.csv$", "_retention_summary.csv", output_file)
retention_tracker <- list()
log_step <- function(step_name, current_n, original_n) {
    pct <- round(100 * current_n / original_n, 2)
    cli_inform(c("i" = "{step_name}: {.val {current_n}} retained ({.val {pct}}%)"))
    retention_tracker[[step_name]] <<- current_n
}
df <- read.csv(input_file, stringsAsFactors = FALSE)
original_n <- nrow(df)
log_step("Original dataset", original_n, original_n)
names(df) <- tolower(names(df))
if (!"species" %in% names(df)) {
    possible_species_cols <- c("scientificname", "taxon", "speciesname", "name", "organismname")
    matched_col <- intersect(possible_species_cols, names(df))[1]
    if (!is.na(matched_col)) {
        df <- df %>% dplyr::rename(species = all_of(matched_col))
        cli_alert_info("Renamed column '{matched_col}' to 'species'")
    }
}
if (!"longitude" %in% names(df) && "long" %in% names(df)) df <- df %>% dplyr::rename(longitude = long)
if (!"latitude" %in% names(df) && "lat" %in% names(df)) df <- df %>% dplyr::rename(latitude = lat)
required_cols <- c("longitude", "latitude", "species")
missing_cols  <- setdiff(required_cols, names(df))
if (length(missing_cols) > 0) {
    cli_abort("Missing required columns: {.val {missing_cols}}")
}
df_clean <- df %>%
```

```r
    dplyr::mutate(longitude = as.numeric(longitude), latitude = as.numeric(latitude)) %>%
    dplyr::filter(!is.na(longitude) & !is.na(latitude))
log_step("Missing coordinates removed", nrow(df_clean), original_n)
df_clean <- df_clean %>%
    dplyr::distinct(longitude, latitude, species, .keep_all = TRUE)
log_step("Duplicate coordinates removed", nrow(df_clean), original_n)
cc_results <- clean_coordinates(
    df_clean,
    lon = "longitude",
    lat = "latitude",
    species = "species",
    tests = c("val", "zeros", "equ", "sea"),
    verbose = FALSE
)
df_valid <- as.data.frame(cc_results) %>%
    dplyr::filter(.summary == TRUE) %>%
    dplyr::select(-starts_with("."))
log_step("CoordinateCleaner passed", nrow(df_valid), original_n)
occ_sf <- st_as_sf(df_valid, coords = c("longitude", "latitude"), crs = 4326, remove = FALSE) %>%
    dplyr::filter(latitude < 66.5 & latitude > -60)
log_step("Arctic/Antarctica excluded", nrow(occ_sf), original_n)
land <- ne_download(scale = 110, type = "land", category = "physical", returnclass = "sf") %>%
    st_transform(4326) %>%
    st_make_valid()
sf_use_s2(FALSE)
occ_on_land <- st_join(
    occ_sf,
    land %>% dplyr::select(geometry),
    join = st_within,
    left = FALSE
)
sf_use_s2(TRUE)
log_step("Ocean records removed", nrow(occ_on_land), original_n)
final_clean <- occ_on_land %>%
    st_drop_geometry() %>%
    dplyr::distinct(longitude, latitude, species, .keep_all = TRUE)
set.seed(123)
thinned_list <- thin(
    loc.data = final_clean,
    lat.col = "latitude",
    long.col = "longitude",
    spec.col = "species",
    thin.par = 20,
    reps = 1,
    locs.thinned.list.return = TRUE,
    write.files = FALSE,
    verbose = FALSE
)
if (length(thinned_list) == 0 || nrow(thinned_list[[1]]) == 0) {
    cli_abort("No occurrences remained after spatial thinning.")
}
final_occ <- thinned_list[[1]] %>%
    dplyr::rename(longitude = Longitude, latitude = Latitude) %>%
    dplyr::distinct(longitude, latitude, .keep_all = TRUE)
log_step("After 20-km spatial thinning", nrow(final_occ), original_n)
```

```r
retention_summary <- data.frame(
    Step = names(retention_tracker),
    Retained = unlist(retention_tracker),
    stringsAsFactors = FALSE
) %>%
    dplyr::mutate(
        Removed = lag(Retained, default = original_n) - Retained,
        Percent_of_Original = round(100 * Retained / original_n, 2)
    )
print(as_tibble(retention_summary))
print(summary(final_occ))
print(skim(final_occ))
cor_matrix <- cor(final_occ %>% dplyr::select(longitude, latitude), use = "complete.obs")
corrplot(cor_matrix, method = "circle", type = "upper")
world <- ne_countries(scale = "medium", returnclass = "sf")
map_plot <- ggplot() +
    geom_sf(data = world, fill = "antiquewhite", color = "gray70", linewidth = 0.25) +
    geom_point(data = final_occ, aes(x = longitude, y = latitude), color = "red", size = 1.8, alpha = 0.8) +
    annotation_scale(location = "bl", width_hint = 0.3) +
    annotation_north_arrow(
        location = "bl", which_north = "true",
        pad_x = unit(0.3, "in"), pad_y = unit(0.3, "in"),
        style = north_arrow_fancy_orienteering
    ) +
    coord_sf(xlim = c(-180, 180), ylim = c(-60, 66.5), expand = FALSE) +
    theme_minimal() +
    labs(
        title = "Cleaned and Spatially Thinned Occurrences",
        subtitle = paste0("20-km spatial thinning | Global (excl. Arctic & Antarctica) | ", nrow(final_occ), " occurrences retained"),
        x = "Longitude", y = "Latitude"
    )
write.csv(final_occ, output_file, row.names = FALSE)
write.csv(retention_summary, summary_file, row.names = FALSE)
cli_alert_success("Process finished! Files saved to:")
cli_li("Occurrences: {.path {output_file}}")
cli_li("Retention Summary: {.path {summary_file}}")

sf_use_s2(TRUE)
occ_path <- "C:/"
occ_data <- read.csv(occ_path)
lon_col <- grep("^lon", colnames(occ_data), ignore.case = TRUE, value = TRUE)[1]
lat_col <- grep("^lat", colnames(occ_data), ignore.case = TRUE, value = TRUE)[1]
if (is.na(lon_col) || is.na(lat_col)) {
    stop("Longitude/Latitude columns could not be identified automatically.")
}
occ_coords <- occ_data[, c(lon_col, lat_col)]
colnames(occ_coords) <- c("longitude", "latitude")
occ_coords <- na.omit(occ_coords)
occ_sf <- st_as_sf(occ_coords, coords = c("longitude", "latitude"), crs = 4326)
climate_dir <- "C:/Users/20106/Desktop/R5 files/wc2.1_10m_bio"
climate_files <- list.files(climate_dir, pattern = "\\.tif$", full.names = TRUE, ignore.case = TRUE)
if (length(climate_files) == 0) {
    stop("No .tif raster files were found in the specified directory.")
}
bioclim_raw <- terra::rast(climate_files)
```

```r
layer_names <- names(bioclim_raw)
num_extract <- as.numeric(gsub("[^0-9]", "", layer_names))
if (!any(is.na(num_extract))) {
    bioclim_raw <- bioclim_raw[[order(num_extract)]]
}

if (nlyr(bioclim_raw) >= 19) {
    bioclim_raw <- bioclim_raw[[1:19]]
} else {
    stop("Fewer than 19 bioclimatic raster layers were found.")
}
global_extent <- ext(-180, 180, -60, 66.5)
env_layers_global <- crop(bioclim_raw, global_extent)
cat("Loaded", nlyr(env_layers_global), "bioclimatic raster layers (BioClim 1-19).\n")
world_land <- ne_countries(scale = "medium", returnclass = "sf") |> st_make_valid()
equal_area_crs <- "EPSG:54034"
occ_sf_proj <- st_transform(occ_sf, crs = equal_area_crs)
pt_buffers_proj <- st_buffer(occ_sf_proj, dist = 100000)
m_buffered_proj <- st_union(pt_buffers_proj)
m_buffered_wgs84 <- st_transform(m_buffered_proj, crs = 4326) |> st_make_valid()
m_points_region <- st_intersection(m_buffered_wgs84, st_union(world_land)) |> st_make_valid()
asia_subset <- world_land[world_land$continent == "Asia", ]
asia_union <- st_union(asia_subset) |> st_make_valid()
m_combined_union <- st_union(st_geometry(m_points_region), st_geometry(asia_union)) |> st_make_valid()
m_region <- st_intersection(m_combined_union, st_union(world_land)) |> st_make_valid()
env_layers_m <- crop(env_layers_global, vect(m_region), mask = TRUE)
world_map <- map_data("world")
map_m_area <- ggplot() +
    geom_polygon(data = world_map, aes(x = long, y = lat, group = group),
                 fill = "#E0E0E0", color = "#FFFFFF", linewidth = 0.2) +
    geom_sf(data = m_region, aes(fill = "Accessible Area M (Buffer + All Asia)"),
            color = "#B2182B", linewidth = 0.5, alpha = 0.4) +
    geom_sf(data = occ_sf, aes(shape = "Occurrences"),
            color = "#2166AC", size = 1.0, alpha = 0.6) +
    scale_fill_manual(values = c("Accessible Area M (Buffer + All Asia)" = "#B2182B"), name = "") +
    scale_shape_manual(values = c("Occurrences" = 16), name = "") +
    coord_sf(xlim = c(st_bbox(m_region)["xmin"] - 3, st_bbox(m_region)["xmax"] + 3),
             ylim = c(st_bbox(m_region)["ymin"] - 3, st_bbox(m_region)["ymax"] + 3),
             expand = FALSE) +
    theme_minimal() +
    theme(
        panel.grid.major = element_line(color = "grey90", linetype = "dotted"),
        legend.position = "bottom",
        legend.box = "horizontal",
        plot.title = element_text(face = "bold", size = 12)
    ) +
    labs(
        title = "Delineation of Accessible Area (M)",
        subtitle = "Combined 100-km Occurrence Buffer & All Asia Continent Extent",
        x = "Longitude",
        y = "Latitude"
    )
occ_env <- terra::extract(env_layers_m, occ_coords)
valid_occ <- complete.cases(occ_env)
occ_coords_clean <- occ_coords[valid_occ, , drop = FALSE]
```

```r
occ_vals_all     <- occ_env[valid_occ, -1, drop = FALSE]
occ_sf_clean <- st_as_sf(occ_coords_clean, coords = c("longitude", "latitude"), crs = 4326)
cor_matrix_all <- cor(occ_vals_all, method = "pearson")
plot_cor_heatmap <- function(cor_mat, title_str) {
    cor_df <- as.data.frame(as.table(cor_mat))
    colnames(cor_df) <- c("Var1", "Var2", "Correlation")
        ggplot(cor_df, aes(x = Var1, y = Var2, fill = Correlation)) +
        geom_tile(color = "white") +
        scale_fill_gradient2(low = "#2166AC", mid = "#F7F7F7", high = "#B2182B",
                             limits = c(-1, 1), name = "Pearson\nCorrelation") +
        geom_text(aes(label = round(Correlation, 2)), size = 2.5, color = "black") +
        theme_minimal() +
        theme(axis.text.x = element_text(angle = 45, hjust = 1, vjust = 1),
              axis.title = element_blank()) +
        labs(title = title_str)
}

vif_res <- vifcor(occ_vals_all, th = 0.7)
selected_vars <- vif_res@results$Variables
env_stack_m <- terra::subset(env_layers_m, selected_vars)
env_stack_global <- terra::subset(env_layers_global, selected_vars)
cat("\n--- Variables retained (r <= 0.7) ---\n")
print(selected_vars)
occ_vals_selected <- occ_vals_all[, selected_vars, drop = FALSE]
cor_matrix_selected <- cor(occ_vals_selected, method = "pearson")
set.seed(42)
bg_points <- spatSample(env_stack_m, size = 10000, method = "random", na.rm = TRUE, xy = TRUE)
bg_coords <- bg_points[, c("x", "y")]
colnames(bg_coords) <- c("longitude", "latitude")
bg_vals   <- bg_points[, -(1:2), drop = FALSE]

target_crs <- "EPSG:3857"
env_stack_proj <- terra::project(env_stack_m, target_crs)

occ_sf_proj <- st_transform(occ_sf_clean, crs = target_crs)
bg_sf_proj  <- st_as_sf(bg_coords, coords = c("longitude", "latitude"), crs = 4326) |>
    st_transform(crs = target_crs)

set.seed(42)
sb <- cv_spatial(
    x = occ_sf_proj,
    column = NULL,
    r = env_stack_proj,
    k = 5,
    size = 600000,
    selection = "random",
    iteration = 100,
    biomod2 = FALSE,
    progress = FALSE
)

occ_folds <- sb$folds_ids

bg_intersects <- st_intersects(bg_sf_proj, sb$blocks)
bg_folds_ids  <- sapply(bg_intersects, function(x) if (length(x) > 0) sbblocksfolds[x[1]] else sample(1:5, 1))
```

```
message("--- Running ENMeval Tuning inside Accessible Area (M) ---")
eval_results <- ENMevaluate(
    occs = as.data.frame(occ_coords_clean),
    envs = env_stack_m,
    bg = as.data.frame(bg_coords),
    algorithm = "maxnet",
    partitions = "user",
    user.grp = list(occs.grp = occ_folds, bg.grp = bg_folds_ids),
    tune.args = list(fc = c("L", "LQ", "LQH"), rm = c(0.5, 1, 2, 3)),
    parallel = FALSE
)

tune_comparison <- eval_results@results[, c("fc", "rm", "auc.val.avg", "auc.diff.avg", "cbi.val.avg", "or.10p.avg", "delta.AICc")]
colnames(tune_comparison) <- c("FC", "RM", "Mean_AUC", "AUC_Diff", "Mean_CBI", "Omission_10p", "delta_AICc")
tune_comparison <- tune_comparison[order(tune_comparison$delta_AICc), ]

cat("\n--- Candidate Model Comparison Table ---\n")
print(tune_comparison)

best_model_idx <- which.min(eval_results@results$delta.AICc)
if (is.na(best_model_idx)) {
    best_model_idx <- which.max(eval_results@results$auc.val.avg)
}

best_fc <- eval_results@results$fc[best_model_idx]
best_rm <- as.numeric(as.character(eval_results@results$rm[best_model_idx]))

cat("\nOptimal Model selected: FC =", best_fc, "| RM =", best_rm, "\n")

pres_vals <- occ_vals_selected

presence_vector <- c(rep(1, nrow(pres_vals)), rep(0, nrow(bg_vals)))
env_data        <- rbind(pres_vals, bg_vals)

k_folds <- 5
cv_results <- data.frame()
spec_grid  <- seq(1, 0, length.out = 200)
sens_matrix <- matrix(NA, nrow = length(spec_grid), ncol = k_folds)
response_curves_df <- data.frame()

for (i in 1:k_folds) {
    train_p <- pres_vals[occ_folds != i, , drop = FALSE]
    test_p  <- pres_vals[occ_folds == i, , drop = FALSE]
    train_bg <- bg_vals[bg_folds_ids != i, , drop = FALSE]
    test_bg  <- bg_vals[bg_folds_ids == i, , drop = FALSE]

    p_vec_fold <- c(rep(1, nrow(train_p)), rep(0, nrow(train_bg)))
    data_fold  <- rbind(train_p, train_bg)

    model_fold <- maxnet(
        p = p_vec_fold,
        data = data_fold,
        f = maxnet.formula(p = p_vec_fold, data = data_fold, classes = tolower(best_fc)),
        regmult = best_rm
```

```
    )

    pred_p <- predict(model_fold, test_p, type = "cloglog")
    pred_a <- predict(model_fold, test_bg, type = "cloglog")
    pred_bg_all <- predict(model_fold, bg_vals, type = "cloglog")

    scores <- c(pred_p, pred_a)
    obs   <- c(rep(1, length(pred_p)), rep(0, length(pred_a)))
    roc_i <- roc(obs, scores, quiet = TRUE)

    interp_sens <- approx(roc_i$specificities, roc_i$sensitivities, xout = spec_grid, rule = 2)$y
    interp_sens[1] <- 0
    interp_sens[length(interp_sens)] <- 1
    sens_matrix[, i] <- interp_sens

    coords_i <- coords(roc_i, "best", ret = c("threshold", "sensitivity", "specificity"))
    thresh <- if (length(coords_ithreshold) > 0)coords_ithreshold[1] else NA
    sens   <- if (length(coords_isensitivity) > 0)coords_isensitivity[1] else NA
    spec   <- if (length(coords_ispecificity) > 0)coords_ispecificity[1] else NA

    omission <- if (!is.na(thresh)) sum(pred_p < thresh) / length(pred_p) else NA
    cbi_eval <- ecospat.boyce(fit = as.numeric(c(pred_p, pred_bg_all)), obs = as.numeric(pred_p), nclass = 0, PEplot = FALSE)
    cbi_val  <- if (!is.null(cbi_eval$cor)) cbi_eval$cor else cbi_eval$Spearman.cor

    cv_results <- rbind(cv_results, data.frame(
        Fold = i, AUC = as.numeric(auc(roc_i)), TSS = as.numeric(sens + spec - 1),
        CBI = as.numeric(cbi_val), Threshold = as.numeric(thresh),
        Sensitivity = as.numeric(sens), Specificity = as.numeric(spec), OmissionRate = as.numeric(omission)
    ))

    for (v in names(env_stack_m)) {
        var_range <- seq(min(env_data[[v]], na.rm=TRUE), max(env_data[[v]], na.rm=TRUE), length.out = 100)
        temp_df <- data.frame(matrix(colMeans(env_data, na.rm=TRUE), nrow = 100, ncol = ncol(env_data), byrow = TRUE))
        colnames(temp_df) <- colnames(env_data)
        temp_df[[v]] <- var_range

        pred_resp <- predict(model_fold, temp_df, type = "cloglog")

        response_curves_df <- rbind(response_curves_df, data.frame(
            Fold = as.factor(i), Variable = v, Value = var_range, Suitability = as.numeric(pred_resp)
        ))
    }
}

metrics_summary <- data.frame(
    Metric = colnames(cv_results)[-1],
    Mean  = colMeans(cv_results[, -1], na.rm = TRUE),
    SE    = apply(cv_results[, -1], 2, function(x) sd(x, na.rm = TRUE) / sqrt(sum(!is.na(x))))
)

cat("\n--- 5-Fold Cross-Validation Metrics Summary ---\n")
print(metrics_summary)

avg_response_df <- aggregate(Suitability ~ Variable + Value, data = response_curves_df, FUN = mean)
sd_response_df  <- aggregate(Suitability ~ Variable + Value, data = response_curves_df, FUN = sd)
```

```
avg_response_dfSD < −sdresponsedfSuitability
avg_response_df$Lower <- pmax(0, avg_response_df$Suitability - avg_response_df$SD)
avg_response_df$Upper <- pmin(1, avg_response_df$Suitability + avg_response_df$SD)

ggplot(avg_response_df, aes(x = Value, y = Suitability)) +
    geom_ribbon(aes(ymin = Lower, ymax = Upper), fill = "blue", alpha = 0.2) +
    geom_line(color = "darkblue", linewidth = 1.1) +
    facet_wrap(~ Variable, scales = "free_x") +
    labs(
        title = "Average Response Curves Across 5-Fold Spatial CV",
        subtitle = "Solid line represents the mean response; shaded band represents ±1 SD across folds",
        x = "Variable Value",
        y = "Suitability (cloglog)"
    ) +
    theme_minimal()

mean_sens <- rowMeans(sens_matrix, na.rm = TRUE)
sd_sens   <- apply(sens_matrix, 1, sd, na.rm = TRUE)
se_sens   <- sd_sens / sqrt(k_folds)
t_val     <- qt(0.975, df = k_folds - 1)

ci_lower  <- pmax(0, mean_sens - t_val * se_sens)
ci_upper  <- pmin(1, mean_sens + t_val * se_sens)

ci_lower[1] <- ci_upper[1] <- mean_sens[1] <- 0
ci_lower[length(ci_lower)] <- ci_upper[length(ci_upper)] <- mean_sens[length(mean_sens)] <- 1

avg_roc_df <- data.frame(
    FalsePositive   = 1 - spec_grid,
    MeanSensitivity = mean_sens,
    LowerCI         = ci_lower,
    UpperCI         = ci_upper
)

ggplot(avg_roc_df, aes(x = FalsePositive, y = MeanSensitivity)) +
    geom_ribbon(aes(ymin = LowerCI, ymax = UpperCI), fill = "lightblue", alpha = 0.4) +
    geom_line(color = "darkblue", linewidth = 1.2) +
    geom_abline(intercept = 0, slope = 1, linetype = "dashed", color = "red") +
    scale_x_continuous(limits = c(0, 1), breaks = seq(0, 1, 0.2), expand = c(0, 0)) +
    scale_y_continuous(limits = c(0, 1), breaks = seq(0, 1, 0.2), expand = c(0, 0)) +
    labs(
        title = "Average ROC Curve (5-Fold Cross-Validation)",
        subtitle = paste("Mean AUC =", round(mean(cv_results$AUC, na.rm = TRUE), 3),
                         "± SE =", round(metrics_summarySE[metricssummaryMetric == "AUC"], 4)),
        x = "1 - Specificity (False Positive Rate)",
        y = "Sensitivity (True Positive Rate)"
    ) +
    theme_minimal() +
    coord_equal()

optimal_maxnet <- maxnet(
    p = presence_vector,
    data = env_data,
    f = maxnet.formula(p = presence_vector, data = env_data, classes = tolower(best_fc)),
```

```
    regmult = best_rm
)

base_pred <- predict(optimal_maxnet, env_data, type = "cloglog")
base_auc  <- as.numeric(auc(roc(presence_vector, as.numeric(base_pred), quiet = TRUE)))

var_imp <- data.frame(Variable = names(env_stack_m), Importance = NA)

set.seed(42)
for (j in seq_along(names(env_stack_m))) {
    v_name <- names(env_stack_m)[j]
    env_perm <- env_data
    env_perm[[v_name]] <- sample(env_perm[[v_name]])

    perm_pred <- predict(optimal_maxnet, env_perm, type = "cloglog")
    perm_auc  <- as.numeric(auc(roc(presence_vector, as.numeric(perm_pred), quiet = TRUE)))

    var_imp$Importance[j] <- max(0, base_auc - perm_auc)
}

total_drop <- sum(var_imp$Importance)
if (total_drop > 0) {
    var_impRelativeImportance_Pct < −(var_impImportance / total_drop) * 100
} else {
    var_imp$RelativeImportance_Pct <- 100 / nrow(var_imp)
}

var_imp <- var_imp[order(var_imp$RelativeImportance_Pct, decreasing = TRUE), ]

cat("\n--- Relative Variable Importance (%) ---\n")
print(var_imp[, c("Variable", "RelativeImportance_Pct")])

ggplot(var_imp, aes(x = reorder(Variable, RelativeImportance_Pct), y = RelativeImportance_Pct)) +
    geom_bar(stat = "identity", fill = "#2B5C8F") +
    coord_flip() +
    labs(
        title = "Relative Variable Importance",
        x = "Bioclimatic Variable",
        y = "Relative Contribution (%)"
    ) +
    theme_minimal()

suitability_map_global <- terra::predict(env_stack_global, optimal_maxnet, type = "cloglog", na.rm = TRUE)

par(mfrow = c(1, 1))

plot(
    suitability_map_global,
    main = "Continuous Habitat Suitability (Global Excl. Arctic & Antarctica)",
    col = terrain.colors(100, rev = TRUE),
    range = c(0, 1)
)
points(occ_coords_clean, pch = 20, cex = 0.4, col = "black")

reclass_matrix <- matrix(c(
```

```r
    0.00, 0.25, 1,
    0.25, 0.50, 2,
    0.50, 0.75, 3,
    0.75, 1.00, 4
), ncol = 3, byrow = TRUE)

classified_map_global <- classify(suitability_map_global, reclass_matrix, include.lowest = TRUE)
custom_colors <- c("#FFFFB2", "#2CA02C", "#FF8C00", "#FF0000")

plot(
    classified_map_global,
    main = "Categorized Habitat Suitability (Global Excl. Arctic & Antarctica)",
    col = custom_colors,
    type = "classes",
    levels = c("Rarely Suitable (0.00–0.25)",
               "Moderately Suitable (>0.25–0.50)",
               "Highly Suitable (>0.50–0.75)",
               "Very Highly Suitable (>0.75–1.00)")
)
points(occ_coords_clean, pch = 20, cex = 0.4, col = "black")

cell_area_km2 <- terra::cellSize(classified_map_global, unit = "km")
area_by_class <- terra::zonal(cell_area_km2, classified_map_global, fun = "sum", na.rm = TRUE)
colnames(area_by_class) <- c("Class_ID", "Area_km2")

class_labels <- data.frame(
    Class_ID = 1:4,
    Suitability_Tier = c("Rarely Suitable (0.00–0.25)",
                         "Moderately Suitable (>0.25–0.50)",
                         "Highly Suitable (>0.50–0.75)",
                         "Very Highly Suitable (>0.75–1.00)")
)

area_summary <- merge(class_labels, area_by_class, by = "Class_ID")
area_summary$Percentage <- (area_summary$Area_km2 / sum(area_summary$Area_km2)) * 100

cat("\n--- Surface Area per Habitat Suitability Tier (Global Projection) ---\n")
print(area_summary[, c("Suitability_Tier", "Area_km2", "Percentage")])


model_maxnet <- optimal_maxnet
global_future_extent <- ext(-180, 180, -60, 66.5)
future_dir <- "C:"
models <- c("IPSL-CM6A-LR", "BCC-CSM2-MR", "MPI-ESM1-2-HR")
ssps   <- c("126", "245", "585")
target_indices <- sapply(selected_vars, function(x) {
    num <- unlist(regmatches(x, gregexpr("[0-9]+", x)))
    as.numeric(num[length(num)])
})
reclass_matrix <- matrix(c(
    0.00, 0.25, 1,
    0.25, 0.50, 2,
    0.50, 0.75, 3,
    0.75, 1.00, 4
), ncol = 3, byrow = TRUE)
```

```
custom_colors <- c("#FFFFB2", "#2CA02C", "#FF8C00", "#FF0000")
class_labels <- data.frame(
    Class_ID = 1:4,
    Suitability_Tier = c(
        "Rarely Suitable (0.00–0.25)",
        "Moderately Suitable (>0.25–0.50)",
        "Highly Suitable (>0.50–0.75)",
        "Very Highly Suitable (>0.75–1.00)"
    )
)

calc_area_summary <- function(pred_cont_raster, label_name) {
    pred_classified <- classify(pred_cont_raster, reclass_matrix, include.lowest = TRUE)
    cell_area_km2   <- terra::cellSize(pred_classified, unit = "km")
    area_by_class   <- terra::zonal(cell_area_km2, pred_classified, fun = "sum", na.rm = TRUE)
    colnames(area_by_class) <- c("Class_ID", "Area_km2")

    df_summary <- merge(class_labels, area_by_class, by = "Class_ID", all.x = TRUE)
    df_summaryArea_km2[is.na(df_summaryArea_km2)] <- 0
    df_summary$Percentage <- (df_summary$Area_km2 / sum(df_summary$Area_km2)) * 100
    df_summary$Scenario   <- label_name
    return(df_summary[, c("Scenario", "Suitability_Tier", "Area_km2", "Percentage")])
}

fut_preds_ssp     <- list()
all_gcm_ssp_preds <- list()
all_area_summaries <- data.frame()

all_future_files <- list.files(future_dir, pattern = "\\.tif$", full.names = TRUE, recursive = TRUE)

if (length(all_future_files) == 0) {
    stop("No .tif raster files were found in '", future_dir, "'. Check path and files.")
}

par(mfrow = c(1, 1))

for (ssp in ssps) {
    ssp_gcm_preds <- list()

    cat("\n==========================================\n")
    cat("Processing SSP Scenario:", ssp, "(2050)\n")
    cat("==========================================\n")

    for (m in models) {
        cat("    -> Reading local raster for GCM:", m, "...\n")

        pattern_str   <- paste0("(?=.*", m, ")(?=.*(?i)ssp-?", ssp, ")")
        matched_files <- all_future_files[grepl(pattern_str, all_future_files, perl = TRUE)]

        if (length(matched_files) == 0) {
            next
        }

        fut_raw <- terra::rast(matched_files[1])

```

```
        if (max(target_indices) <= nlyr(fut_raw)) {
            fut_sub <- fut_raw[[target_indices]]
        } else {
            stop("File for ", m, " (SSP ", ssp, ") has ", nlyr(fut_raw),
                 " layers, but requires index ", max(target_indices))
        }

        names(fut_sub) <- selected_vars
        fut_sub <- crop(fut_sub, global_future_extent)

        pred_cont <- terra::predict(fut_sub, model_maxnet, type = "cloglog", na.rm = TRUE)
        scenario_label <- paste0(m, "_SSP", ssp)

        ssp_gcm_preds[[m]]            <- pred_cont
        all_gcm_ssp_preds[[scenario_label]] <- pred_cont

        pred_classified <- classify(pred_cont, reclass_matrix, include.lowest = TRUE)

        plot(pred_cont,
             main = paste(m, "| SSP", ssp, "(2050) - Continuous Global"),
             col  = terrain.colors(100, rev = TRUE), range = c(0, 1))

        plot(pred_classified,
             main   = paste(m, "| SSP", ssp, "(2050) - Categorized Global"),
             col    = custom_colors,
             type   = "classes",
             levels = class_labels$Suitability_Tier)

        gcm_summary <- calc_area_summary(pred_cont, scenario_label)
        all_area_summaries <- rbind(all_area_summaries, gcm_summary)
    }

    if (length(ssp_gcm_preds) == 0) next

    ssp_avg_cont         <- mean(rast(ssp_gcm_preds))
    fut_preds_ssp[[ssp]] <- ssp_avg_cont
    ssp_label            <- paste0("Ensemble_SSP", ssp)

    ssp_avg_classified <- classify(ssp_avg_cont, reclass_matrix, include.lowest = TRUE)

    plot(ssp_avg_cont,
         main  = paste("Ensemble Avg Continuous (0-1) | SSP", ssp, "(2050)"),
         col   = terrain.colors(100, rev = TRUE), range = c(0, 1))

    plot(ssp_avg_classified,
         main   = paste("Ensemble Avg Categorized | SSP", ssp, "(2050)"),
         col    = custom_colors,
         type   = "classes",
         levels = class_labels$Suitability_Tier)

    ssp_summary <- calc_area_summary(ssp_avg_cont, ssp_label)
    all_area_summaries <- rbind(all_area_summaries, ssp_summary)
}

if (length(all_gcm_ssp_preds) > 0) {
```

```
    grand_future_stack    <- terra::rast(all_gcm_ssp_preds)
    grand_overall_cont    <- terra::mean(grand_future_stack)
    grand_overall_class   <- classify(grand_overall_cont, reclass_matrix, include.lowest = TRUE)

    plot(grand_overall_cont,
         main = "Grand Overall Future Average Continuous (0-1) (2050)",
         col  = terrain.colors(100, rev = TRUE), range = c(0, 1))

    plot(grand_overall_class,
         main   = "Grand Overall Future Average Categorized (2050)",
         col    = custom_colors,
         type   = "classes",
         levels = class_labels$Suitability_Tier)

    grand_summary <- calc_area_summary(grand_overall_cont, "Grand_Overall_Ensemble")
    all_area_summaries <- rbind(all_area_summaries, grand_summary)

    cat("\n--- Complete Surface Area Summaries for All 13 Scenarios (km²) ---\n")
    print(all_area_summaries)
}


model_maxnet <- optimal_maxnet
baseline_suitability <- suitability_map_global
bin_threshold <- 0.236
cat("Using model cross-validation mean threshold:", bin_threshold, "\n")

global_extent <- ext(-180, 180, -60, 66.5)

future_dir <- "C:/"

models <- c("IPSL-CM6A-LR", "BCC-CSM2-MR", "MPI-ESM1-2-HR")
ssps   <- c("126", "245", "585")

target_indices <- sapply(selected_vars, function(x) {
    num <- unlist(regmatches(x, gregexpr("[0-9]+", x)))
    as.numeric(num[length(num)])
})

all_future_files <- list.files(future_dir, pattern = "\\.tif$", full.names = TRUE, recursive = TRUE)

if (length(all_future_files) == 0) {
    stop("No .tif raster files were found in '", future_dir, "'.")
}

ind_preds <- list()
ssp_preds <- list()

for (ssp in ssps) {
    ssp_model_preds <- list()

    for (m in models) {
        pattern_str <- paste0("(?=.*", m, ")(?=.*(?i)ssp-?", ssp, ")")
        matched_files <- all_future_files[grepl(pattern_str, all_future_files, perl = TRUE)]

```

```
        if (length(matched_files) == 0) {
            next
        }

        fut_raw <- terra::rast(matched_files[1])

        if (max(target_indices) <= nlyr(fut_raw)) {
            fut_sub <- fut_raw[[target_indices]]
        } else {
            stop("File for ", m, " (SSP ", ssp, ") has ", nlyr(fut_raw),
                 " layers, but requires index ", max(target_indices))
        }

        names(fut_sub) <- selected_vars
        fut_sub <- crop(fut_sub, global_extent)

        pred_cont <- terra::predict(fut_sub, model_maxnet, type = "cloglog", na.rm = TRUE)

        model_key <- paste0(m, "_SSP", ssp)
        ind_preds[[model_key]] <- pred_cont
        ssp_model_preds[[m]]   <- pred_cont
    }

    if (length(ssp_model_preds) > 0) {
        ssp_preds[[paste0("SSP", ssp, "_Ensemble")]] <- mean(rast(ssp_model_preds))
    }
}

overall_future_avg <- mean(rast(ind_preds))

current_bin <- baseline_suitability >= bin_threshold

change_pal  <- c("lightgrey", "red", "green", "darkblue")
change_labs <- c("Unsuitable", "Loss", "Gain", "Stable")

analysis_maps <- c(
    ind_preds,
    ssp_preds,
    list(Grand_Overall_Ensemble = overall_future_avg)
)

stats_list <- list()

for (name in names(analysis_maps)) {
    fut_bin <- analysis_maps[[name]] >= bin_threshold

    change_map_raw <- current_bin + (2 * fut_bin) + 1
    change_map     <- change_map_raw

    cell_areas <- terra::cellSize(change_map, unit = "km")
    map_vals   <- terra::values(change_map, mat = FALSE)
    area_vals  <- terra::values(cell_areas, mat = FALSE)

    stats_df <- data.frame(Class = change_labs, Class_Val = 1:4)
    stats_dfArea_km2 < −sapply(stats_dfClass_Val, function(v) {
```

```
        sum(area_vals[map_vals == v], na.rm = TRUE)
    })

    total_valid_area    <- sum(stats_df$Area_km2)
    stats_dfPercentage < −(stats_dfArea_km2 / total_valid_area) * 100
    stats_df$Scenario   <- name

    stats_list[[name]]  <- stats_df

    cat("\n===================================\n")
    cat("Global Spatial Shift Statistics (2050):", name, "\n")
    cat("===================================\n")
    print(stats_df[, c("Class", "Area_km2", "Percentage")])

    change_df <- as.data.frame(change_map, xy = TRUE, na.rm = TRUE)
    colnames(change_df)[3] <- "Change_Class"
    change_dfChangeclass < −factor(change_dfChange_Class, levels = 1:4, labels = change_labs)

    gg_change <- ggplot() +
        geom_tile(data = change_df, aes(x = x, y = y, fill = Change_Class)) +
        scale_fill_manual(values = change_pal, name = "Classification") +
        labs(
            title = paste("Global Species Range Shift (2050):", name),
            subtitle = "2041–2060 vs Current Conditions (Excl. Arctic & Antarctica)",
            x = "Longitude (degrees)",
            y = "Latitude (degrees)"
        ) +
        theme_minimal() +
        coord_equal() +
        theme(
            legend.position = "right",
            legend.text     = element_text(size = 10),
            plot.title      = element_text(size = 13, face = "bold"),
            axis.title      = element_text(size = 11, face = "bold")
        )

    print(gg_change)
}

all_range_shifts <- do.call(rbind, stats_list)
```
