# Supplementary material for: Global Habitat Suitability Modeling of the Giant Honeybee (Apis dorsata) Under Future Climate Change Scenarios
Source: Insects. 2026 Sep 12;17(9):954. doi: 10.3390/insects17090954 (PMC13607631; doi:10.3390/insects17090954)
Supplement: Supplementary file 1 [file insects-17-00954-s001.zip › File S3.pdf]

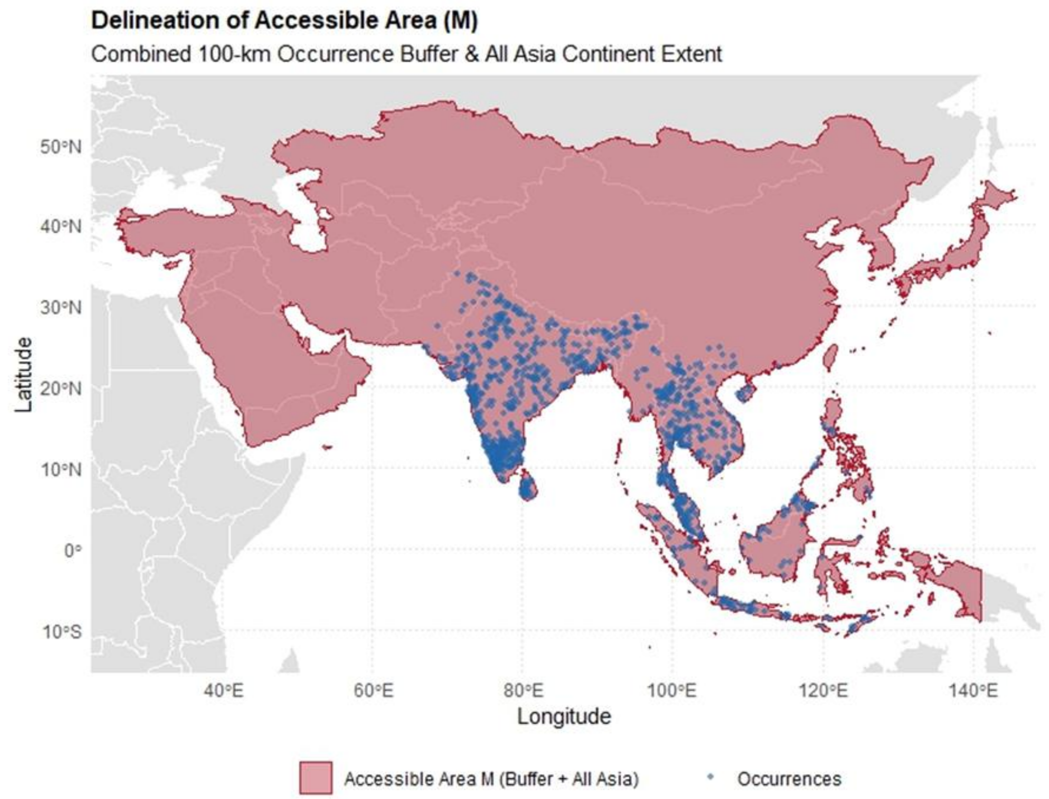

Supplementary Figure S1. Delineation of the accessible area (*M*) used for model calibration.

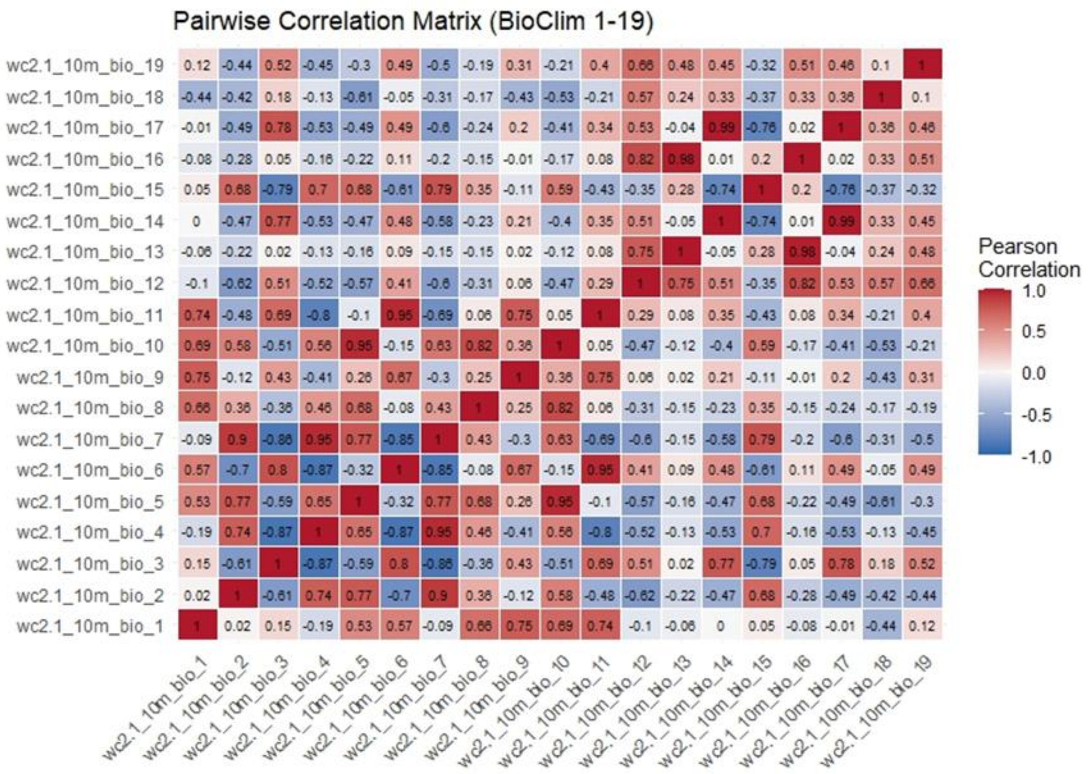

Supplementary Figure S2. Heatmap showing pairwise Pearson correlation coefficients (r) among the 19 WorldClim bioclimatic variables. Colors indicate the strength and direction of correlations, ranging from dark blue (r = −1.0) to dark red (r = 1.0).

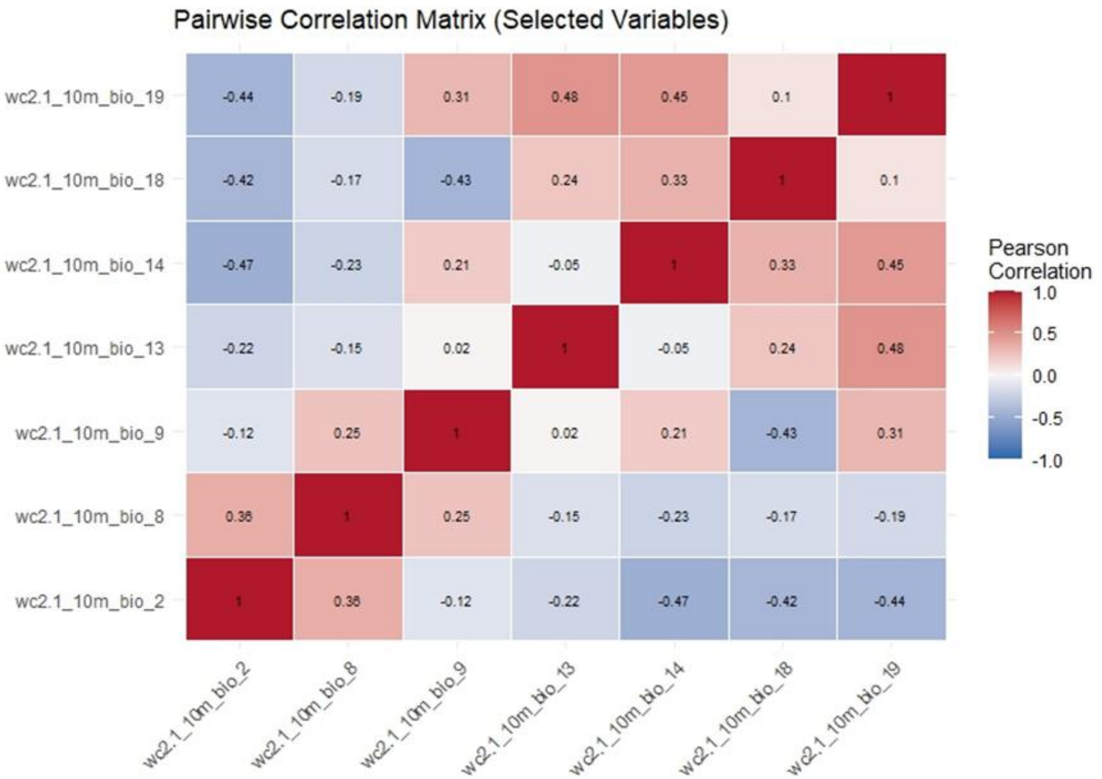

Supplementary Figure S3. Heatmap displaying pairwise Pearson correlation coefficients (r) among the final subset of non-collinear WorldClim predictors (bio2, bio8, bio9, bio13, bio14, bio18, and bio19).

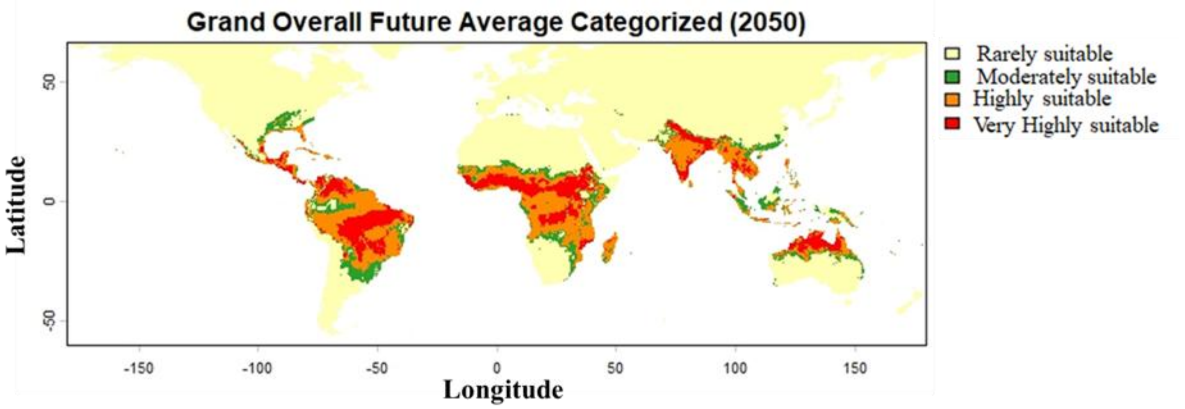

Supplementary Figure S4. Grand overall future average projections of environmental suitability in 2050 across three models (IPSL-CM6A-LR, BCC-CSM2-MR, and MPI-ESM1-2-HR) and three SSPs (SSP126, SSP245, and SSP585).

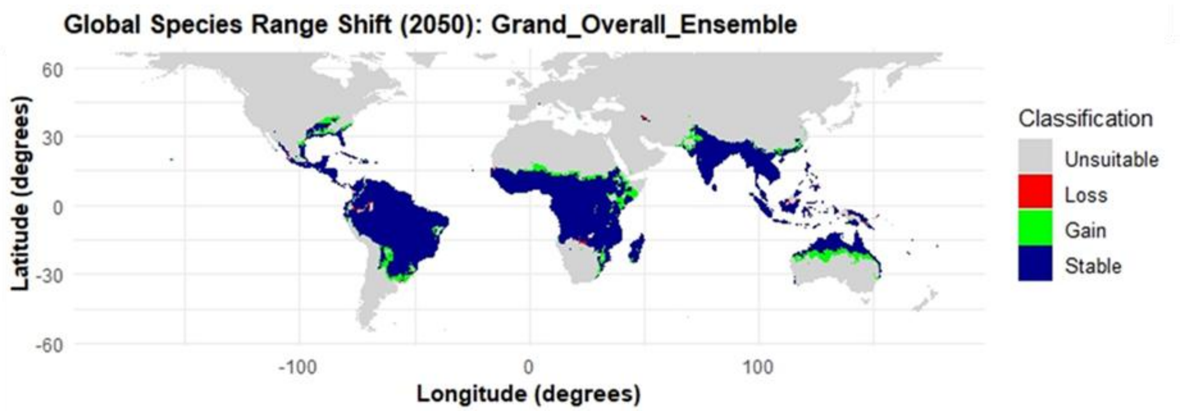

Supplementary Figure S5. Global range shifts (2050) under current and future conditions. The future projection for 2050 represents the grand overall future average of environmental suitability across three climate models (IPSL-CM6A-LR, BCC-CSM2-MR, and MPI-ESM1-2-HR) and three SSPs (SSP126, SSP245, and SSP585).
