# Supplementary material for: Global Habitat Suitability Modeling of the Giant Honeybee (Apis dorsata) Under Future Climate Change Scenarios
Source: Insects. 2026 Sep 12;17(9):954. doi: 10.3390/insects17090954 (PMC13607631; doi:10.3390/insects17090954)
Supplement: Supplementary file 1 [file insects-17-00954-s001.zip › File s4.pdf]

**Supplementary Table S1.** Comparison of candidate MaxEnt models generated through ENMeval tuning within the accessible area (*M*).

| Rank | Feature Class (FC) | Regularization Multiplier (RM) | Mean AUC | ΔAICc |
|---|---|---|---|---|
| 1 | LQH | 0.5 | 0.9048 | 0.00 |
| 2 | LQH | 1.0 | 0.9031 | 37.98 |
| 3 | LQH | 2.0 | 0.8999 | 88.61 |
| 4 | LQH | 3.0 | 0.8976 | 128.89 |
| 5 | LQ | 0.5 | 0.8880 | 384.23 |
| 6 | LQ | 1.0 | 0.8860 | 464.13 |
| 7 | LQ | 2.0 | 0.8783 | 644.53 |
| 8 | LQ | 3.0 | 0.8734 | 760.75 |
| 9 | L | 0.5 | 0.8539 | 1058.08 |
| 10 | L | 1.0 | 0.8539 | 1060.84 |
| 11 | L | 2.0 | 0.8538 | 1067.29 |
| 12 | L | 3.0 | 0.8538 | 1073.03 |

**Supplementary Table S2.** Future (2050) projection of *Apis dorsata* habitat suitability area (%) under different climate scenarios and models.

| Scenario | Climate model | Rarely Suitable | Moderately Suitable | Highly Suitable | Very Highly Suitable |
|---|---|---|---|---|---|
| SSP126 | IPSL-CM6A-LR | 67.35 | 7.15 | 16.39 | 9.11 |
| SSP126 | BCC-CSM2-MR | 66.62 | 6.34 | 15.29 | 11.75 |
| SSP126 | MPI-ESM1-2-HR | 68.24 | 6.84 | 15.99 | 8.93 |
| SSP126 | Ensemble | 67.01 | 7.24 | 16.17 | 9.58 |
| SSP245 | IPSL-CM6A-LR | 66.94 | 7.32 | 16.34 | 9.40 |
| SSP245 | BCC-CSM2-MR | 66.13 | 6.23 | 15.59 | 12.05 |
| SSP245 | MPI-ESM1-2-HR | 68.21 | 6.95 | 16.74 | 8.10 |
| SSP245 | Ensemble | 66.61 | 7.33 | 16.62 | 9.44 |
| SSP585 | IPSL-CM6A-LR | 66.09 | 7.06 | 16.31 | 10.54 |
| SSP585 | BCC-CSM2-MR | 65.35 | 6.45 | 15.79 | 12.41 |
| SSP585 | MPI-ESM1-2-HR | 67.23 | 6.84 | 18.38 | 7.55 |
| SSP585 | Ensemble | 65.86 | 7.14 | 16.98 | 10.02 |
| Overall | Grand Overall Ensemble | 66.45 | 7.27 | 16.61 | 9.67 |

**Supplementary Table S3.** Percentage of global spatial shifts in habitat suitability of *Apis dorsata* between current conditions and 2050.

| Scenario | Climate model | Unsuitable (%) | Loss (%) | Gain (%) | Stable (%) |
|---|---|---|---|---|---|
| SSP126 | IPSL-CM6A-LR | 66.64 | 0.34 | 2.75 | 30.27 |
| SSP126 | BCC-CSM2-MR | 65.48 | 0.80 | 3.90 | 29.82 |
| SSP126 | MPI-ESM1-2-HR | 67.52 | 0.34 | 1.86 | 30.28 |
| SSP126 | Ensemble | 66.35 | 0.30 | 3.03 | 30.32 |
| SSP245 | IPSL-CM6A-LR | 66.18 | 0.38 | 3.20 | 30.24 |
| SSP245 | BCC-CSM2-MR | 64.95 | 0.84 | 4.43 | 29.78 |
| SSP245 | MPI-ESM1-2-HR | 67.53 | 0.33 | 1.86 | 30.28 |
| SSP245 | Ensemble | 65.95 | 0.32 | 3.43 | 30.30 |
| SSP585 | IPSL-CM6A-LR | 65.29 | 0.43 | 4.10 | 30.18 |
| SSP585 | BCC-CSM2-MR | 64.19 | 0.80 | 5.19 | 29.82 |
| SSP585 | MPI-ESM1-2-HR | 66.63 | 0.30 | 2.76 | 30.31 |
| SSP585 | Ensemble | 65.23 | 0.26 | 4.15 | 30.36 |
| Overall | Grand Overall Ensemble | 65.84 | 0.26 | 3.55 | 30.35 |
