# Supplementary material for: Global Habitat Suitability Modeling of the Giant Honeybee (Apis dorsata) Under Future Climate Change Scenarios
Source: Insects. 2026 Sep 12;17(9):954. doi: 10.3390/insects17090954 (PMC13607631; doi:10.3390/insects17090954)
Supplement: Supplementary file 1 [file insects-17-00954-s001.zip › File S1.pdf]

No	longitude	latitude
1	107.0019	14.21002
2	100.9412	21.8517
3	100.7748	5.201363
4	79.22767	23.52853
5	116.553	6.210847
6	100.4863	6.320965
7	85.1135	25.59806
8	85.52026	24.32466
9	90.99819	26.2995
10	79.26807	10.98944
11	72.58371	23.20292
12	75.25899	31.30345
13	78.32822	11.27544
14	102.4114	15.0071
15	100.8172	5.614296
16	74.87968	30.19116
17	83.9915	28.18806
18	97.99013	19.25582
19	101.1188	6.230322
20	78.71474	11.08149
21	76.67677	10.72163
22	88.36081	21.78934
23	75.1487	20.39597
24	72.82264	19.42378
25	98.45874	8.9981
26	101.1283	19.89219
27	111.8469	2.3401
28	123.1761	9.355017
29	109.5108	18.22186
30	77.4829	16.74156
31	74.80201	13.83283
32	100.0248	22.17891
33	103.0159	14.92253
34	73.68505	24.60286
35	81.60819	21.20576
36	110.3755	19.51991
37	100.811	15.81299
38	72.65017	23.48925
39	98.78529	9.011495
40	102.0309	14.82275
41	99.48291	14.07067
42	100.4738	14.58247

43	105.6114	22.39901
44	74.63483	32.88804
45	78.98	17.912
46	104.8615	24.75367
47	101.4191	14.57544
48	73.9161	20.10151
49	77.90448	26.65647
50	77.59593	8.426557
51	97.10101	17.47744
52	99.59735	22.64511
53	103.0436	22.65244
54	116.9163	4.791523
55	105.84	9.77002
56	74.922	13.45057
57	105.815	15.11405
58	79.74144	11.77113
59	80.11178	21.37271
60	103.192	-4.06861
61	101.1722	-0.10221
62	74.89417	25.08151
63	112.2818	2.459355
64	106.2044	17.5453
65	100.3961	-1.01957
66	73.01872	26.29829
67	77.57675	12.83082
68	80.22714	26.50553
69	98.30198	8.11485
70	81.8376	25.40904
71	101.7067	3.642252
72	77.74396	29.01321
73	102.4616	1.251022
74	75.74006	11.34181
75	108.6731	19.06372
76	98.21339	18.74837
77	77.79298	11.40155
78	76.56369	25.85155
79	80.56485	7.233851
80	69.26571	23.37782
81	76.11525	13.02244
82	114.6312	3.986313
83	101.3848	4.467892
84	100.6541	18.17885
85	98.91873	8.049983

86	125.3788	-8.67597
87	77.62648	11.54229
88	103.9362	11.21418
89	75.51568	24.84423
90	118.8299	10.08054
91	73.66165	20.0087
92	101.54	3.559578
93	119.4019	11.14974
94	80.18383	21.81287
95	118.7358	9.814434
96	75.71581	27.16557
97	99.79134	19.14538
98	91.41688	24.38408
99	83.05988	21.59706
100	78.06234	28.17485
101	119.152	10.39917
102	100.1658	-0.00282
103	79.80866	13.28781
104	107.0669	12.32302
105	74.9168	12.87153
106	100.0746	20.36783
107	79.31928	21.82741
108	99.1356	18.75095
109	98.24311	8.629017
110	80.78669	6.978407
111	106.5033	9.813831
112	99.95307	19.29157
113	103.5025	14.92157
114	76.46035	15.33616
115	88.97033	22.45802
116	80.82782	6.772057
117	108.123	15.76603
118	79.99738	19.35877
119	81.80405	17.01023
120	98.58336	14.71902
121	97.57385	24.63314
122	72.4631	22.42532
123	103.1527	19.43069
124	77.25159	13.22761
125	76.21007	10.82657
126	121.2047	14.56822
127	71.84654	23.31941
128	71.96193	29.31165

129	100.7788	13.61224
130	83.14628	19.90645
131	73.01919	33.11554
132	77.4745	9.495646
133	102.2545	16.80124
134	89.61528	22.29691
135	78.64124	12.28087
136	101.8468	3.400997
137	101.6464	0.116822
138	81.63987	17.2472
139	99.20956	2.489044
140	100.7822	5.413225
141	73.47437	17.56214
142	76.0566	18.23863
143	100.5272	13.90186
144	101.6384	12.65652
145	102.3304	18.99595
146	102.5856	18.94123
147	77.34922	10.34757
148	77.17956	9.559042
149	106.7925	10.49218
150	106.7945	10.88632
151	99.16903	10.40696
152	76.26692	26.26135
153	100.0767	15.77919
154	78.63556	15.22309
155	76.00129	11.77878
156	75.92047	22.52045
157	77.39177	8.410942
158	73.86919	15.7303
159	79.09823	10.76395
160	92.75161	24.6865
161	88.50599	26.58407
162	101.2831	12.68297
163	75.17548	27.64968
164	76.94522	11.21838
165	80.11394	28.95097
166	117.803	4.962755
167	79.94211	18.85007
168	82.57529	25.14711
169	98.3575	17.79586
170	96.09457	21.97959
171	100.616	14.80551

172	77.12221	28.65482
173	76.59801	27.59682
174	72.90782	20.92297
175	73.74623	17.40039
176	103.8957	22.30701
177	103.1011	21.43242
178	75.5915	12.01205
179	102.7231	0.331794
180	74.2987	18.34482
181	102.2655	1.172962
182	83.78767	19.3542
183	115.0299	-8.53461
184	86.64222	22.12207
185	102.8298	16.42946
186	118.5079	5.209593
187	77.01217	10.81962
188	79.64164	22.76761
189	76.58349	9.981136
190	75.41172	25.30265
191	78.62343	11.81736
192	81.88604	20.96216
193	77.61245	22.94102
194	107.5322	-6.88997
195	99.87116	7.676454
196	99.98554	12.41335
197	114.9951	-8.19525
198	119.9741	-9.69934
199	116.4287	5.828389
200	73.47227	25.11595
201	78.30756	12.3878
202	107.412	-7.13079
203	101.008	18.71334
204	79.24512	21.64952
205	74.99332	13.12278
206	76.92029	10.54046
207	73.42499	18.44196
208	105.2622	-5.51985
209	71.38339	34.00904
210	99.09507	19.74624
211	109.3544	-0.06976
212	114.8453	-3.44521
213	80.39709	8.349631
214	103.3141	17.686

215	72.67915	21.40116
216	107.4617	-6.50501
217	101.1705	4.309298
218	73.58041	18.23525
219	108.3273	14.66141
220	76.06867	22.97293
221	78.04702	11.64227
222	78.2962	18.83697
223	100.1031	13.77461
224	75.69486	18.23833
225	76.6053	27.00758
226	78.28029	18.06205
227	77.13615	11.48294
228	92.34481	24.35268
229	76.72512	10.93475
230	100.7716	22.00008
231	77.32324	13.74005
232	69.77638	23.39431
233	74.87694	31.64428
234	79.60177	12.53516
235	76.60008	30.49701
236	79.21414	13.8122
237	78.66043	12.58799
238	77.83834	22.43284
239	77.15096	28.42584
240	112.2557	-7.81015
241	93.73158	25.53802
242	100.752	18.5601
243	75.6835	24.98197
244	78.55411	14.75262
245	73.7754	33.44835
246	117.0509	-0.38227
247	100.3537	-0.24174
248	70.6221	21.50775
249	115.6873	-1.71729
250	101.2556	5.836981
251	84.2593	25.21846
252	85.10336	19.66645
253	78.54311	26.9341
254	84.36567	18.9678
255	77.13718	8.681657
256	76.38991	21.262
257	80.80787	7.779544

258	76.98886	11.60864
259	101.4023	4.016808
260	80.97748	7.962425
261	104.3577	-4.346
262	106.8628	-6.39559
263	78.57986	10.88724
264	108.1593	16.10967
265	79.44829	14.18849
266	76.6137	21.28117
267	73.87271	15.51463
268	70.2673	21.6411
269	75.39005	12.15202
270	107.2009	-6.80394
271	102.3203	13.48946
272	105.6489	-5.76649
273	75.95087	21.07345
274	78.08561	10.4148
275	75.88535	17.62663
276	91.78486	22.49181
277	114.8914	4.953347
278	103.9025	16.23769
279	120.2792	14.74206
280	76.88368	28.67007
281	108.783	15.36354
282	110.2868	-7.83992
283	79.61653	10.81624
284	77.58409	13.17065
285	102.9605	4.402967
286	101.7819	14.72723
287	76.66718	11.86964
288	92.0304	24.14826
289	100.7397	4.859953
290	98.46406	19.29659
291	77.32257	28.14009
292	104.9814	14.67731
293	103.6143	14.45562
294	98.94437	2.451662
295	106.8914	-6.18338
296	86.44658	23.78976
297	81.28195	6.281205
298	77.76486	12.86116
299	101.9323	2.428552
300	102.3492	2.41128

301	95.22092	28.35089
302	101.5872	21.62315
303	83.0787	19.70681
304	107.5288	11.58377
305	106.6349	10.6707
306	101.964	16.25494
307	95.06553	28.61431
308	90.26905	23.87906
309	106.9776	10.54923
310	74.94572	12.6182
311	98.50462	18.6287
312	100.3218	13.79349
313	123.9668	-9.69521
314	82.64841	18.0357
315	106.3709	15.39018
316	79.86903	20.59807
317	101.7781	3.231081
318	76.15667	26.05286
319	101.1862	14.67114
320	89.0012	25.28929
321	76.84983	28.18342
322	93.69366	27.79161
323	75.96761	12.43025
324	79.98654	19.81257
325	110.489	-7.77358
326	87.8169	22.55596
327	101.0024	22.76778
328	85.46931	23.4379
329	98.80926	18.48301
330	80.05076	12.81643
331	100.7324	18.85643
332	107.4279	11.42126
333	74.28695	16.36415
334	100.8446	13.99974
335	100.2981	5.34825
336	79.84749	11.13183
337	72.62732	23.79176
338	76.36762	9.774595
339	104.4842	18.17814
340	77.2197	31.11139
341	75.83804	25.23186
342	100.4399	7.47643
343	99.73977	19.64184

344	92.39802	27.10397
345	78.03189	12.61843
346	75.68288	20.70838
347	73.06991	19.04732
348	88.02116	23.5339
349	102.5544	2.039358
350	99.8618	8.174299
351	78.99889	29.4193
352	88.88538	21.88424
353	111.143	-7.60899
354	77.2088	11.19618
355	73.69511	17.9304
356	118.0378	5.413406
357	78.83538	18.10517
358	77.67487	10.85363
359	99.39756	13.44821
360	75.94303	12.15146
361	81.05652	16.70164
362	80.62567	7.598286
363	70.09936	22.47856
364	99.38351	19.06925
365	77.11894	30.86256
366	75.529	31.3217
367	105.2743	14.44224
368	95.36883	27.57307
369	87.23378	23.4051
370	74.59664	16.85867
371	108.9888	12.10886
372	126.1978	6.351411
373	86.09197	20.47229
374	72.99057	33.51569
375	78.53911	19.67621
376	72.87736	20.70479
377	126.2592	6.918229
378	85.44493	25.26441
379	78.07426	30.28309
380	125.7912	7.351898
381	73.70531	16.11579
382	74.07276	16.21892
383	75.38494	11.94468
384	90.40136	24.40873
385	75.32139	17.66789
386	74.02909	16.74158

387	99.89522	20.03822
388	102.5617	3.747944
389	92.65542	23.73353
390	79.43659	13.6571
391	78.27472	17.51299
392	101.8544	3.793983
393	106.5976	16.7949
394	103.0614	17.02941
395	103.7075	1.358233
396	77.05708	28.92717
397	89.47328	21.98033
398	115.4009	-8.26633
399	80.22365	13.11714
400	116.4519	5.444004
401	99.75792	15.32298
402	100.9374	4.765899
403	80.12358	23.23235
404	75.5612	21.30603
405	100.9638	22.10389
406	95.02559	27.4438
407	74.37984	23.56068
408	71.44186	23.41465
409	75.461	26.31048
410	86.10447	23.11952
411	73.08062	33.75902
412	102.2529	17.92441
413	120.999	14.67057
414	80.56682	7.033259
415	101.4023	6.059361
416	75.34343	13.27387
417	121.5435	14.01385
418	99.5101	15.07047
419	81.62058	28.06064
420	79.13734	18.46636
421	73.88593	17.94901
422	74.55607	19.11204
423	81.3037	24.53625
424	92.00957	25.2072
425	117.3716	5.771057
426	76.07315	27.04535
427	81.64091	24.11845
428	77.07865	20.5123
429	123.8401	-10.2535

430	100.0199	19.83297
431	99.74366	19.85401
432	69.6828	21.73283
433	76.59478	28.78836
434	88.39374	22.47199
435	78.39409	22.46323
436	93.7298	26.6023
437	120.0829	15.4606
438	88.19011	22.51737
439	100.9535	19.13619
440	69.66705	23.1811
441	81.01012	21.09785
442	124.8645	1.318805
443	103.403	4.029296
444	96.61903	5.128261
445	98.7122	16.60218
446	109.5385	18.72715
447	99.65197	12.69951
448	103.2511	16.24744
449	101.029	14.52293
450	90.78275	23.58655
451	80.49205	24.99524
452	99.38113	20.04704
453	102.1709	19.97721
454	119.6082	-4.92514
455	72.33621	30.03693
456	110.4444	-7.38684
457	109.2334	12.22486
458	77.69786	17.71916
459	98.92447	18.74903
460	105.158	20.44754
461	75.03307	26.62738
462	67.5872	24.16918
463	74.20245	19.56043
464	106.9904	13.68213
465	99.94431	13.4509
466	74.124	15.29933
467	110.3556	-7.15766
468	101.1543	4.711717
469	117.9903	4.969647
470	106.2847	9.556372
471	101.903	12.56675
472	77.24707	21.59435

473	96.61199	22.10719
474	83.02197	25.37804
475	83.47638	20.68193
476	78.49215	15.47468
477	76.0835	11.60388
478	114.8726	-1.69793
479	74.74798	19.09483
480	79.7907	23.46445
481	76.79452	11.3439
482	79.99709	13.28306
483	75.49195	21.08747
484	105.7527	9.307457
485	93.62623	27.04444
486	100.0681	7.735173
487	100.9752	16.68703
488	101.347	13.75056
489	77.31831	11.3888
490	101.712	17.57892
491	90.91481	26.62599
492	91.54773	26.00144
493	76.35754	11.9724
494	80.97123	26.84597
495	76.89071	28.45833
496	93.31223	27.89059
497	103.593	2.636704
498	78.07057	17.6503
499	93.72237	27.60152
500	101.8613	2.947178
501	76.59873	11.32286
502	99.02907	10.10783
503	79.87866	23.13989
504	110.2162	-1.46457
505	71.46507	22.64261
506	81.07676	22.17597
507	79.91064	24.88839
508	79.31965	14.02052
509	105.5474	15.58198
510	84.29903	27.61783
511	100.8766	22.54221
512	116.0236	5.803045
513	101.1256	16.3899
514	79.34515	12.89644
515	83.52261	20.19283

516	100.4011	13.60372
517	101.6947	3.015878
518	101.9126	2.703861
519	110.5615	-7.20457
520	81.35094	21.19383
521	85.66986	19.91033
522	101.0442	4.523215
523	111.6495	1.028335
524	105.5985	20.34837
525	96.8446	3.9558
526	107.9149	22.44609
527	108.0031	13.94515
528	106.6512	-6.29462
529	114.8153	4.052651
530	74.27536	18.96008
531	105.696	-5.07967
532	101.707	2.832431
533	73.11378	26.46965
534	110.114	-7.7665
535	81.52519	24.65468
536	78.0766	10.96008
537	101.2542	3.333227
538	118.9445	5.32983
539	77.74584	13.0645
540	74.98551	15.44006
541	80.04872	23.03644
542	74.48425	15.82097
543	81.32005	8.37352
544	79.43866	23.8323
545	80.51312	8.010142
546	98.92977	19.39879
547	115.1822	-8.19463
548	91.89736	26.13235
549	78.76275	19.10079
550	100.5164	7.00495
551	77.36335	23.23231
552	81.63776	25.20446
553	78.20192	26.20179
554	114.1079	22.38373
555	85.8756	20.27224
556	102.6388	5.003962
557	75.63587	25.4488
558	81.45319	19.9258

559	78.18513	12.73699
560	110.1613	1.501732
561	76.12022	10.4429
562	87.45526	23.46368
563	99.98812	19.57172
564	102.0546	14.60315
565	81.66703	19.59477
566	99.87265	8.644075
567	87.68523	23.67765
568	72.07441	23.8372
569	78.14896	29.95251
570	72.42378	23.68459
571	100.8862	6.152178
572	76.98575	9.576177
573	124.2122	-9.65758
574	102.402	4.383058
575	79.46788	11.15194
576	119.8912	-1.20343
577	99.41078	8.807392
578	76.35546	10.02525
579	99.51199	19.33273
580	100.9099	6.391462
581	98.69913	18.63931
582	91.43677	26.4412
583	76.54675	12.52732
584	88.1511	24.23085
585	81.71355	17.59834
586	117.4806	5.34504
587	77.34476	28.65227
588	110.9028	-6.65101
589	76.564	32.129
590	77.60214	12.04603
591	102.1323	4.646129
592	79.0747	12.22528
593	75.69237	12.46418
594	100.6565	16.57875
595	90.20331	26.94621
596	109.4817	19.49914
597	99.4016	13.87955
598	102.4069	4.636008
599	100.6574	5.716324
600	98.6255	8.9012
601	101.7992	4.062844

602	101.3191	5.452949
603	86.96799	23.67346
604	96.1187	16.88559
605	79.38757	20.27179
606	76.66198	12.37749
607	68.88727	23.69578
608	75.6307	12.98914
609	73.76054	24.42999
610	103.7955	22.82073
611	90.90951	26.89557
612	85.77319	26.42872
613	108.0541	12.67966
614	85.70625	20.17592
615	78.39382	17.32109
616	76.9861	20.6843
617	98.11727	3.59644
618	83.49676	27.17655
619	78.45388	13.63633
620	98.30753	8.810403
621	108.3382	12.46442
622	75.6519	12.24538
623	85.39901	20.23007
624	117.8875	4.397931
625	74.21075	16.68396
626	76.93126	27.83777
627	73.2774	22.39505
628	106.2165	24.7992
629	76.12151	12.59568
630	102.6061	17.96559
631	71.21842	21.60449
632	77.06721	10.05727
633	106.1715	11.38142
634	77.3742	28.40899
635	99.45993	12.53699
636	99.07092	17.67782
637	72.86704	21.29613
638	77.66227	27.09489
639	102.7963	5.624781
640	98.64847	9.958494
641	76.46252	26.11189
642	105.2528	16.54029
643	80.03582	24.43097
644	98.86936	18.93972

645	76.36825	10.53028
646	102.4629	17.68865
647	70.75336	21.00353
648	105.5109	15.39845
649	80.41243	6.450317
650	105.813	10.07527
651	95.76422	27.43943
652	78.65071	26.76919
653	115.2588	-8.51585
654	102.288	14.35282
655	99.60894	15.44078
656	118.1706	5.598349
657	73.47825	21.22861
658	102.7965	17.49506
659	73.5988	21.39126
660	80.55375	28.81274
661	99.6283	7.700849
662	106.7233	-6.55395
663	67.29416	24.92742
664	96.53581	27.44547
665	76.81469	9.785169
666	98.3196	19.44576
667	120.4391	-8.60611
668	100.6784	21.57995
669	97.64817	3.687233
670	98.66884	8.593918
671	79.84183	7.244868
672	101.9944	19.75108
673	101.2058	14.34042
674	102.1151	14.98476
675	104.7539	17.95447
676	102.587	20.52689
677	74.25918	22.85129
678	76.3881	27.24298
679	100.275	21.72592
680	80.59167	22.29583
681	73.18404	19.27244
682	103.867	13.41247
683	77.14064	11.99639
684	108.3917	22.7951
685	79.83875	10.79968
686	98.91899	8.237477
687	73.75699	18.36561

688	111.4146	2.078203
689	76.94494	12.81599
690	75.68662	21.09765
691	106.9121	-6.59709
692	72.50241	23.0019
693	101.442	12.99413
694	100.3679	22.02271
695	87.71109	22.38221
696	75.86647	11.20006
697	77.03212	14.29258
698	98.3412	7.935753
699	95.61122	27.25603
700	87.01091	25.2615
701	98.1364	24.1843
702	77.70016	27.58145
703	108.173	23.71994
704	83.4637	27.6481
705	73.42513	18.76073
706	104.94	21.13987
707	93.51021	26.70449
708	103.5872	19.71303
709	81.02327	23.72221
710	107.9049	-6.77859
711	95.24494	21.6164
712	98.0127	18.50909
713	73.23237	19.92412
714	95.22006	20.91714
715	73.41456	19.22555
716	85.37417	25.02398
717	76.45301	12.35119
718	73.76115	19.07553
719	82.68589	22.41153
720	93.17112	26.57753
721	99.24505	8.916221
722	72.86616	19.96566
723	103.5977	11.78839
724	106.985	22.51597
725	73.74043	19.5908
726	96.20447	27.4858
727	103.4687	11.18718
728	71.06296	21.00721
729	71.21449	22.43315
730	101.4827	21.4289

731	79.60231	13.74871
732	73.89489	17.71164
733	83.27941	27.4832
734	76.0129	10.62235
735	77.20703	30.49923
736	102.2522	12.97209
737	115.1573	4.549138
738	100.337	15.02134
739	101.5274	14.08787
740	80.83069	21.27786
741	121.0836	-8.71446
742	72.90914	21.48351
743	101.7358	17.91149
744	79.95019	7.42734
745	91.21206	26.75834
746	116.4506	6.403645
747	89.4598	22.40594
748	100.2582	16.818
749	114.4734	-2.18261
750	82.07443	19.05465
751	76.95583	11.01685
752	77.30837	12.95938
753	125.5445	-8.54362
754	73.48167	30.46956
755	105.9159	13.98541
756	78.67088	10.40508
757	81.99162	25.90952
758	78.26317	10.06823
759	80.79771	6.406304
760	100.699	23.50448
761	109.1744	-6.97501
762	74.55118	26.48346
763	103.8964	1.86227
764	77.76657	12.67497
765	72.11022	21.57705
766	116.7046	6.046657
767	102.8199	5.104714
768	97.99482	3.863396
769	79.28403	11.74248
770	78.90422	10.3595
771	73.45781	22.62034
772	100.9273	12.79161
773	85.69293	26.75933

774	101.8785	0.429939
775	107.2375	10.96556
776	100.2399	6.947236
777	78.10369	22.54967
778	101.3998	14.36546
779	81.22988	8.53991
780	73.29912	24.21827
781	90.40231	23.73646
782	76.28793	10.21902
783	98.38263	9.297581
784	110.3503	1.540975
785	77.86192	12.38352
786	102.3952	22.30327
787	109.1463	19.0691
788	102.7294	2.145878
789	98.70443	19.13268
790	99.96366	12.72828
791	79.05481	29.83783
792	76.46468	11.77876
793	79.37295	25.13925
794	80.7488	8.034772
795	70.57055	21.12436
796	73.0184	19.52717
797	102.0345	15.98072
798	94.40285	16.79868
799	99.30656	9.100092
800	102.5021	0.247548
801	101.4569	5.582786
802	101.9758	4.10615
803	79.82786	28.90329
804	115.4691	4.693414
805	104.1654	12.05822
806	77.37464	12.28238
807	92.51608	26.20413
808	78.08406	27.92067
809	79.87243	6.919392
810	104.8445	16.63489
811	76.38898	28.15805
812	100.6067	14.33266
813	108.8826	18.75784
814	79.3318	29.28723
815	99.65618	12.91031
816	80.14223	8.278735

817	97.95776	19.49099
818	97.57865	24.44781
819	99.65829	8.744672
820	73.898	18.51173
821	101.0203	12.96223
822	99.36521	12.82387
823	81.91951	21.85167
824	77.29887	28.87594
825	99.10135	8.119181
826	73.48251	18.98896
827	103.7855	13.7729
828	80.67603	23.32272
829	77.1403	13.37401
830	101.5737	16.73995
831	118.7198	5.214422
832	81.64679	21.39217
833	85.38336	26.96728
834	103.9872	13.55311
835	99.87984	20.37599
836	77.60664	12.25438
837	81.05029	6.857129
838	86.11583	26.81603
839	94.35987	26.66598
840	91.64253	26.19267
841	76.64516	12.19134
842	77.2456	10.57813
843	99.39818	18.31506
844	77.07807	12.59342
845	75.50734	13.97656
846	87.52434	25.13162
847	80.64518	27.58397
848	87.00627	22.98834
849	78.64881	10.15383
850	81.53479	20.71518
851	87.23691	26.67328
852	88.5245	22.96384
853	98.96719	14.32694
854	99.42125	1.35567
855	96.84249	20.00748
856	105.6882	10.49356
857	88.38052	22.72608
858	75.88346	10.98029
859	81.24121	28.47779

860	101.1456	-1.61009
861	102.7826	18.06337
862	110.1436	19.98274
863	108.6803	12.18259
864	104.4445	-2.48175
865	123.934	-10.039
866	76.70934	30.67804
867	107.7698	-6.9288
868	77.01611	11.8282
869	102.1582	3.567839
870	80.57995	6.524645
871	78.94385	14.53293
872	80.46691	16.65113
873	80.03904	6.824749
874	77.75495	30.44825
875	111.4674	1.203737
876	118.1745	4.91257
877	108.2138	-7.39624
878	118.361	5.401846
879	74.52436	15.08909
880	80.35761	7.478309
881	101.2818	20.82698
882	75.50796	11.74577
883	125.213	-8.96931
884	78.53331	9.98186
885	105.731	18.43819
886	101.636	6.500356
887	78.53397	17.49023
888	77.63616	12.51573
889	77.8649	22.05506
890	93.97813	26.70991
891	107.591	16.46559
892	81.42288	27.11557
893	79.13078	12.8788
894	106.6316	23.90098
895	76.64	11.53108
896	101.7603	5.800373
897	74.23886	24.00163
898	74.36839	31.50866
899	78.97361	17.48135
900	101.9034	14.46937
901	101.6149	3.364399
902	109.7383	-7.12045

903	82.71599	21.6366
904	86.16875	22.81965
905	78.88713	12.5996
906	76.41732	11.5654
907	95.407	27.226
908	81.70595	22.6801
909	77.68371	13.37218
910	101.9577	6.021511
911	77.9967	10.19424
912	100.9247	13.808
913	102.6755	17.30501
914	73.77208	18.64924
915	78.32199	30.13187
916	125.9682	-8.63012
917	89.54834	26.64734
918	82.09596	21.11242
919	104.8456	11.33587
920	98.35149	8.369076
921	79.73682	12.09622
922	91.40371	25.87597
923	94.29665	25.01089
924	100.6057	13.54877
925	80.52631	6.82726
926	102.2596	2.864055
927	88.76235	22.09062
928	72.68355	25.13294
929	87.31988	22.42566
930	75.81019	26.87056
931	68.75503	27.52941
932	72.73379	21.12857
933	75.1055	13.42348
934	79.65582	29.46042
935	103.9531	1.550965
936	86.76759	21.93031
937	78.80733	17.66297
938	81.71312	20.83206
939	126.3664	-8.85444
940	99.81265	7.440509
941	75.5227	32.38968
942	102.2747	2.18985
943	114.5116	-8.14882
944	75.77183	23.19633
945	99.17803	9.550961

946	85.60381	25.99311
947	78.33568	13.08696
948	107.9294	-7.0665
949	78.36591	12.78847
950	102.243	12.53946
951	75.17912	20.02389
952	76.41601	12.66546
953	106.7609	-6.86525
954	77.37247	11.09636
955	74.87029	32.71809
956	78.64413	17.32208
957	101.0654	13.22946
958	78.04832	27.1719
959	108.0915	-7.52964
960	108.5275	-6.75643
961	117.3745	2.863094
962	76.59966	10.17207
963	99.61935	18.13452
964	76.57499	9.385273
965	75.34331	19.87617
966	101.0251	13.61267
967	70.80216	22.30386
968	109.2453	-7.40486
969	76.91754	12.32386
970	73.79053	18.14081
971	77.77578	23.34448
972	100.3401	18.23372
973	75.59042	28.36217
974	82.08832	22.29797
975	82.19906	22.06407
976	83.31248	21.819
977	77.54804	10.26278
978	78.8687	10.89362
979	77.43965	13.40593
980	76.19594	10.6114
981	78.8031	17.1813
982	100.5981	14.07111
983	83.32125	17.86628
984	100.5431	5.364674
985	79.24536	11.31888
986	73.82346	19.89766
987	73.73703	19.33865
988	78.2132	11.8062

989	77.76917	11.90733
990	73.05384	22.2472
991	71.64396	24.78011
992	99.86636	8.412085
993	103.9483	1.363861
994	82.60148	18.59938
995	101.2629	21.9145
996	99.81511	2.099846
997	72.17185	21.77724
998	106.9074	14.56153
999	82.05826	20.829
1000	91.19051	23.42845
1001	78.86246	29.58448
1002	77.80316	29.94221
1003	76.4534	10.98093
1004	101.5118	3.095866
1005	112.9346	2.009777
1006	103.621	1.576104
1007	79.05603	21.18707
1008	76.95998	10.33022
1009	78.9445	13.15111
1010	73.32528	19.45011
1011	99.8681	12.22535
1012	106.5311	-6.739
1013	88.63466	24.36259
1014	87.05038	23.24247
1015	77.25931	10.18497
1016	90.47221	23.54852
1017	103.4402	11.68161
1018	101.3774	-2.08024
1019	73.27099	18.99007
1020	76.37802	13.2705
1021	73.58262	18.54383
1022	77.96429	13.12309
1023	79.91999	6.684368
1024	89.39689	26.84946
1025	72.86768	19.04659
1026	72.97493	19.27029
1027	81.70006	19.2072
1028	75.86792	13.16232
1029	80.75733	7.321045
1030	99.26581	18.58686
1031	76.502	22.838

1032	87.85498	22.98676
1033	78.12415	9.91468
1034	76.1268	11.17897
1035	102.9471	1.840392
1036	80.67628	16.51976
1037	80.22851	12.89383
1038	79.49376	29.0249
1039	86.82014	20.71414
1040	100.3765	19.66379
1041	116.0944	5.968884
1042	89.93452	22.81408
1043	74.93373	13.65385
1044	76.51109	9.597662
1045	79.54779	18.00696
1046	73.26576	22.09605
1047	67.0316	24.8537
1048	103.1275	11.51306
1049	80.24483	7.709052
1050	105.3247	14.11715
1051	80.31231	7.180499
1052	106.1016	15.19463
1053	102.6509	20.75839
1054	90.04439	23.24561
1055	75.27602	12.49291
1056	82.84235	21.28042
1057	93.93684	24.81701
1058	104.5413	13.78573
1059	102.096	20.72011
1060	84.49911	27.57525
